# Modulation of miR-146b by *N6*-methyladenosine modification remodels tumor-associated macrophages and enhances anti-PD-1 therapy in colorectal cancer

**DOI:** 10.1007/s13402-023-00839-0

**Published:** 2023-07-05

**Authors:** Shuying He, Wen Song, Shudan Cui, Jiating Li, Yonghong Jiang, Xueqing Chen, Liang Peng

**Affiliations:** 1 Department of Gastroenterology, First Affiliated Hospital of Guangzhou Medical University, Guangzhou Medical University, No. 151, Yanjiang West Road, Guangzhou City, 510120 Guangdong Province China; 2 Dongguan People’s Hospital, Dongguan City, Guangdong Province China

**Keywords:** Colorectal cancer, miR-146b, Tumor-associated macrophages, PD-L1, *N*6-methyladenosine, PD-1

## Abstract

**Purpose:**

MicroRNA-146b (miR-146b) alleviates experimental colitis in mice by mediating macrophage polarization and the release of inflammatory factors. Our goals were to evaluate the antitumor efficacy of miR-146b in colorectal cancer (CRC) and to investigate the underlying mechanisms.

**Methods:**

We used murine models of CRC to evaluate whether miR-146b influenced the progression of tumors independent of tumor-associated macrophages (TAMs). RNA immunoprecipitation, *N*6-methyladenosine (m^6^A) RNA immunoprecipitation and in vitro pri-miRNA processing assays were conducted to examine whether m^6^A mediates the maturation of pri-miR-146b/miR-146b. In a series of in vitro and in vivo experiments, we further defined the molecular mechanisms of methyltransferase-like 3 (METTL3)/miR-146b-mediated antitumor immunity and its efficacy in combination with anti-PD-1 immunotherapy.

**Results:**

We found that miR-146b deletion supported tumor progression by increasing the number of alternatively activated (M2) TAMs. Mechanistically, the m^6^A-related “writer” protein METTL3 and “reader” protein HNRNPA2B1 controlled miR-146b maturation by regulating the m^6^A modification region of pri-miR-146b. Furthermore, miR-146b deletion promoted the polarization of M2-TAMs by enhancing phosphoinositide 3-kinase (PI3K)/AKT signaling, and this effect was mediated by the class IA PI3K catalytic subunit p110β, which reduced T cell infiltration, aggravated immunosuppression and ultimately promoted tumor progression. METTL3 knockdown or miR-146b deletion induced programmed death ligand 1 (PD-L1) production via the p110β/PI3K/AKT pathway in TAMs and consequently augmented the antitumor activity of anti-PD-1 immunotherapy.

**Conclusions:**

The maturation of pri-miR-146b is m^6^A-dependent, and miR-146b deletion-mediated TAM differentiation promotes the development of CRC by activating the PI3K/AKT pathway, which induces upregulation of PD-L1 expression, inhibits T cell infiltration into the TME and enhances the antitumor activity of anti-PD-1 immunotherapy. The findings reveal that targeting miR-146b can serve as an adjuvant to anti-PD-1 immunotherapy.

**Graphical Abstract:**

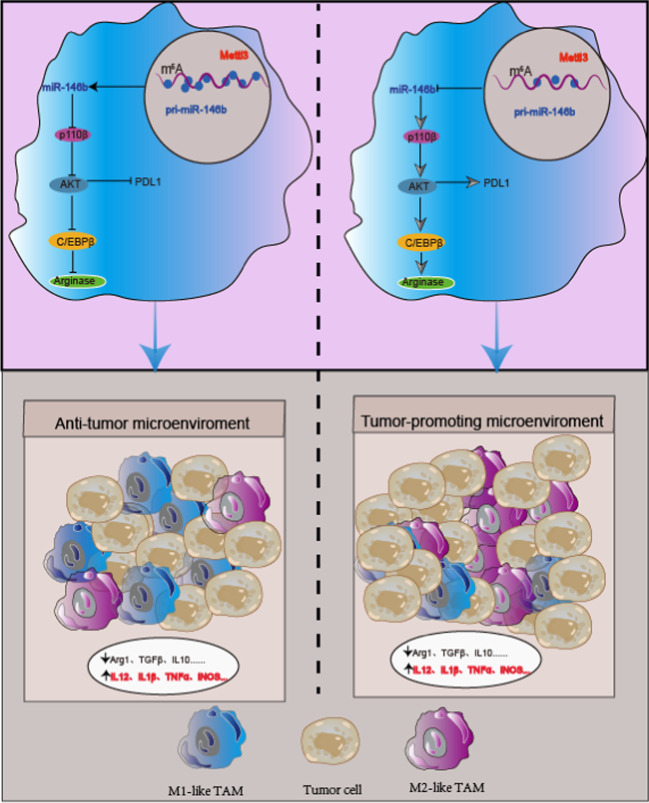

**Supplementary Information:**

The online version contains supplementary material available at 10.1007/s13402-023-00839-0.

## Background

Colorectal cancer (CRC) is one of the most lethal human malignancies in the world [1]. In recent years, immunotherapy has rapidly developed and benefits some cancer patients by affecting immune checkpoints, including the cytotoxic T lymphocyte associated protein 4 (CTLA-4) or the programmed cell death 1 (PD-1) signaling pathway [2, 3], which is a promising approach to activate antitumor immunity and can improve T cell function and reduce tumor burden by strengthening cytotoxic CD8^+^ T cell (CTL) infiltration to slow tumor progression and prolong patient survival. However, the majority of CRC patients with mismatch-repair-proficiency and microsatellite instability-low (pMMR-MSI-L) tumors are unresponsive to treatment [4, 5]. Thus, the mechanism of immunotherapy resistance must be elucidated, and novel markers to predict immunotherapy resistance need to be identified.

Tumor-associated macrophages (TAMs) are critical drivers of the immunosuppressive microenvironment and are recruited to tumors by colony stimulating factor (CSF), transforming growth factor (TGF-β1) and chemokines, such as CCL2 (MCP-1) [6], and these cells mostly exhibit an alternatively activated macrophage (M2)-like phenotype [7]. M2-TAMs can directly stimulate tumor cell proliferation and invasion and suppress T cell recruitment and activation through the production of cytokines, such as interleukin (IL)-10 or epidermal growth factor (EGF)[8]. Hence, modifying the properties and function of TAMs in tumors can enhance antitumor immune responses. In addition, resistance to immunotherapies can be mediated by abundant infiltration of tumor-associated myeloid cells, including macrophages [9]. However, accumulating evidence suggests that programmed death ligand 1 (PD-L1)^+^ TAMs in tumors could better predict a patient’s response to immunotherapy than PD-L1^−^ TAMs in non-small-cell lung cancer patients [10, 11]. These data suggested that PD-L1^+^ TAMs were related to anti-PD-1/PD-L1 therapy resistance in the tumor microenvironment (TME). A better understanding of the mechanism by which PD-L1 regulates immune checkpoints in TAMs is necessary [12, 13].

MicroRNAs (miRNAs) widely participate in macrophage polarization in the TME, and their aberrant expression could affect the balance of macrophage polarization [14]. Evidence from our laboratory has demonstrated that microRNA-146b (miR-146b) inhibits tumor progression in a colitis-associated cancer (CAC) mouse model by targeting TAMs with miR-146b mimic-containing nanoparticles [13], but the mechanism remains unclear. In the current study, we demonstrated that *N*6-methyladenosine (m^6^A) is a key regulator that modifies the transcription of pri-miR-146b to mature miR-146b in macrophages. MiR-146b deficiency increased the number of M2-TAMs accompanied by the upregulation of PD-L1 expression through PI3K/AKT pathway activation, which inhibited T cell infiltration in the TME and resulted in immune suppression and tumor development. Importantly, the combination of miR-146b depletion and a PD-1/PD-L1 checkpoint inhibitor was shown to enhance the antitumor activity of effector immune cells. In summary, our findings revealed the mechanism by which miR-146b functions in colorectal cancer to promote PD-1 blockade, thereby providing potential new biomarkers and a therapeutic avenue for tumors refractory to ICI treatment.

## Materials and methods

### Mouse husbandry

WT C57BL/6 mice (6–8 weeks old) were purchased from Guangdong Medical Laboratory Animal Center (Guangzhou, China), and miR-146b deficient (*miR-146b*^*−/−*^) mice on a C57BL/6 background were housed in the Guangzhou Medical University Animal Experiment Center (Guangzhou, China). All animal protocols were approved by the Animal Ethics Committee of Guangzhou Medical University. The animals were maintained under specific pathogen-free conditions in the Guangzhou Medical University Animal Experiment Center (Guangzhou, China).

### Tumor studies

Six- to eight-week-old female or male WT and *miR-146b*^*−/−*^ mice were implanted with 5 × 10^5^ MC38 cells in 100 μl of phosphate-buffered saline (PBS) by subcutaneous injection in the right flank, and tumor growth was monitored for up to 21 days. Tumor volumes were determined by measuring the length (*l*) and width (*w*) and were calculated as follows: tumor volume = (*l*^2^ x *w*)/2. All in vivo monoclonal antibodies (mAbs) were from BioXCell (New Hampshire, USA). Animals were euthanized before the maximum IACUC allowable tumor burden of 2 cm^3^/mouse was exceeded [12]. Tumor dimensions were measured 2 times per week beginning on day 7.

For the in vivo depletion study, mice were pretreated by an intraperitoneal injection (i.p.) with anti-CD8 mAbs (200 μg/mouse), anti-NK1.1 mAbs (200 μg/mouse), clodronate (CLO, 1 mg/mouse) or control liposomes (Ctrl, 1 mg/mouse) (Clodronateliposomes.com, Amsterdam, The Netherlands) for 1 week (twice/week) and then subcutaneously injected with MC38 cells, after which the mice were treated with mAbs or CLO for an additional three weeks.

For miR-146b agomir administration, 6- to 8-week-old *miR-146b*^*−/−*^ mice were implanted with 5 × 10^5^ MC38 cells, and after 1 week, the mice were intraperitoneally administered 10 mg/kg miR-146b agomir twice per week, while the control group was administered the same dose of NC for 2 weeks.

In vivo macrophage adoptive transfer experiments were performed. Primary bone marrow-derived macrophages (BMDMs) were polarized to the M2 phenotype as described in the Supplemental Material. M2 cells were mixed 1:1 with MC38 tumor cells, and a total of 1 × 10^6^ cells were injected subcutaneously. Primary BMDMs were polarized to the M2 phenotype, and macrophage-conditioned medium (CM) was harvested, centrifuged at 800 × g for 10 min and filtered through a 0.22-μm syringe filter (Millipore, Billerica, MA, USA). Then, CM mixed with 5 × 10^5^ MC38 tumor cells was injected subcutaneously, and inoculated mice were further treated by intradermal injection with conditioned medium at 3 and 6 days post inoculation.

For inhibitor treatment, *miR-146b*^*−/−*^ BMDMs were pretreated with inhibitors of AKT (GSK2141795, 30 μM), phosphoinositide 3-kinase (PI3K) (LY294002, 20 μM) and p110β (TGX221, 10 μM) for 30 min, polarized to the M2 phenotype, and then mixed with MC38 tumor cells. In some experiments, morpholino (MO) was dissolved in PBS, and WT BMDMs were preincubated with MO–nc (5 μM) and MO-*methyltransferase-like 3* (*Mettl3*) (5 μM) for 12 h, polarized to the M2 phenotype, and then mixed with MC38 tumor cells. All adoptive transfer experiments were carried out in new WT mice.

For anti-PD-1 mAb treatment, 5 × 10^5^ MC38 cells were subcutaneously injected into the flanks of the mice. Tumors were allowed to grow for seven days, and then the mice were treated i.p. with 200 μg of anti-PD-1 mAb (Clone J110) or rat IgG2a isotype control every 3 days for 2 weeks. In the colitis-associated cancer model, mice were intraperitoneally injected with 10 mg/kg azoxymethane (AOM, Sigma-Aldrich Corp, St Louis, MO, USA). One week later, the mice were administered 2% dextran sodium sulphate (DSS, MW 40000–50,000) (MP Biomedicals, Solon, Ohio, USA) in distilled water for 5 days, followed by 14 days of normal drinking water [13]. This cycle was repeated three times. After 5 weeks, the mice were administered 200 μg of anti-PD-1 mAbs or Rat IgG2a isotype control every 3 days for the indicated time. The mice were sacrificed after the third cycle ended.

## Results

### Deletion of miR-146b promotes colon tumor growth by reshaping TAMs

**Fig. 1:**
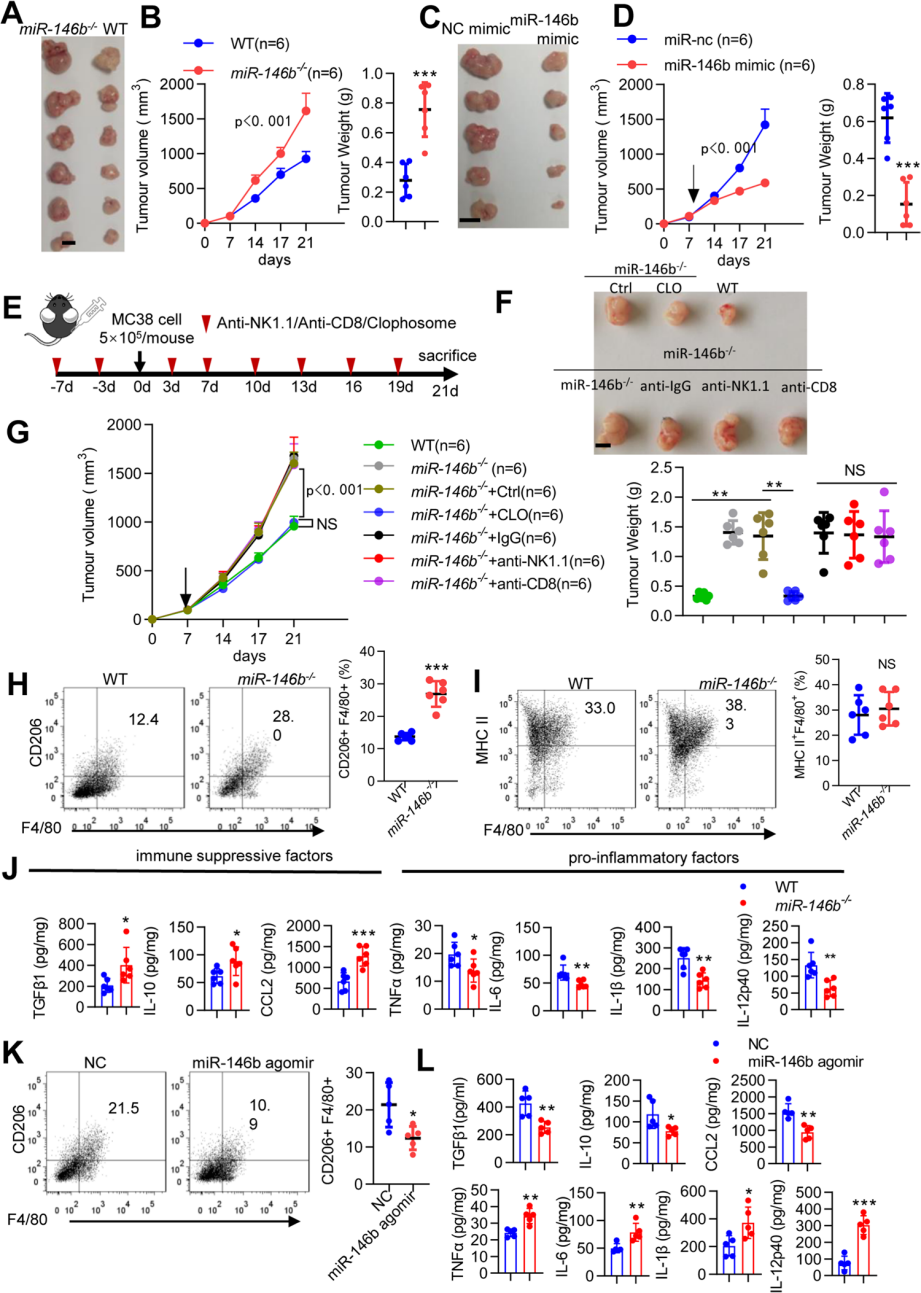
miR146b^−/−^ mice exhibit tumor progression in a macrophage-dependent manner. **(A-B)** WT and *miR-146b*^*−/−*^ mice were implanted with 5 × 10^5^ MC38 cells for 3 weeks. Tumor growth and weight were monitored. **(C-D)** *miR-146b*^*−/−*^ mice were implanted with 5 × 10^5^ MC38 cells for 1 week and then treated with miR-nc or miR-146b mimic intraperitoneally twice weekly. Tumor growth and weight were monitored. **(E)** Mouse models and treatment strategy employed. **(F-G)** WT or *miR-146b*^*−/−*^ mice were pretreated with clodronate and CD8- or NK-depleting antibody every 3 days for 1 week and then injected subcutaneously with MC38 cells. Tumor growth and weight were monitored. n, number of mice. **(H-I)** Flow cytometry analysis of CD11b^+^Gr1^−^F4/80^+^CD206^+^ M2-TAMs and CD11b^+^Gr1^−^F4/80^+^MHC II^+^ M1-TAMs in tumors from WT and miR-146b^−/−^ mice (*n*= 6). **(J)** Protein expression of cytokines in tumors from WT and miR-146b^−/−^ mice. **(K)** Flow cytometry analysis of CD11b^+^Gr1^−^F4/80^+^CD206^+^ M2 TAMs in tumors from miR-146b^−/−^ mice treated with NC or miR-146b agomir intraperitoneally twice a week (n= 6). **(L)** Protein expression of cytokines in tumors from miR-146b^−/−^ mice treated with miR-nc or miR-146b mimic

To explore whether miR-146b was involved in regulating tumor progression, the colon tumor cell line MC38 was subcutaneously inoculated into WT and *miR-146b*^*−/−*^ mice, and after 3 weeks, *miR-146b*^*−/−*^ mice showed markedly increased tumor development and much larger tumor volumes and tumor weights than WT mice (Fig. 1A-B). Consistently, tumor growth was significantly delayed in *miR-146b*^*−/−*^ mice treated with the miR-146b mimic compared with *miR-146b*^*−/−*^ mice treated with miR-nc (Fig. 1C-D). To confirm the contribution of macrophages to the protumor response in *miR-146b*^*−/−*^ mice and exclude the potential role of CD8^+^ T cells and NK cells in *miR-146b*^*−/−*^ mice, *miR-146b*^*−/−*^ mice were pretreated with anti-CD8 antibodies, anti-NK1.1 antibodies or clodronate liposomes to deplete CD8^+^ T cells, NK cells and macrophages respectively (Fig. 1E and Supplementary Fig. 1A-C) [12]. Antibody-mediated depletion of CD8^+^ T and NK cells had no effect on tumor growth in *miR-146b*^*−/−*^ mice (Fig. 1F-G). In contrast, after clodronate liposome treatment, the protumor response in *miR-146b*^*−/−*^ mice was completely abrogated in the absence of macrophages (Fig. 1F-G), indicating that TAMs were essential for tumor development in a miR-146b-deficient host.

Next, we evaluated the effect of miR-146b deficiency on TAM numbers and differentiation in MC38-bearing mice. MiR-146b deficiency switched the activation state of macrophages from an inflammatory M1-like (CD11b^+^Gr1^−^F4/80^+^MHC II^+^) phenotype to a more immunosuppressive M2-like (CD11b^+^Gr1^−^F4/80^+^CD206^+^) phenotype in the TME (Supplementary Fig. 1D and Fig. 1H-I). We further examined the anti-inflammatory and proinflammatory factors in tumors. MiR-146b deficiency increased M2-TAM-related immunosuppressive factors (TGFβ1, IL-10 and CCL2) and inhibited the expression of proinflammatory factors (TNFα, IL-6, IL-1β and IL-12p40) (Fig. 1J). We subsequently analyzed the TAMs in *miR-146b *^*−/−*^ mice treated with the miR-146b mimic, and observed that M2-TAMs were significantly decreased in tumor tissue after treatment with the miR-146b mimic compared with miR-nc (Fig. 1K). Additionally, miR-146b mimic treatment stimulated immune response protein expression (TNFα, IL-6, IL-1β and IL-12p40) and inhibited immunosuppressive protein expression (TGFβ1, IL-10 and CCL2) (Fig. 1L). These data indicated that miR-146b deficiency promoted colon tumor development associated with M2-TAMs in the TME.

### MiR-146b deficiency in macrophages promotes CRC cell proliferation and metastasis

**Fig. 2:**
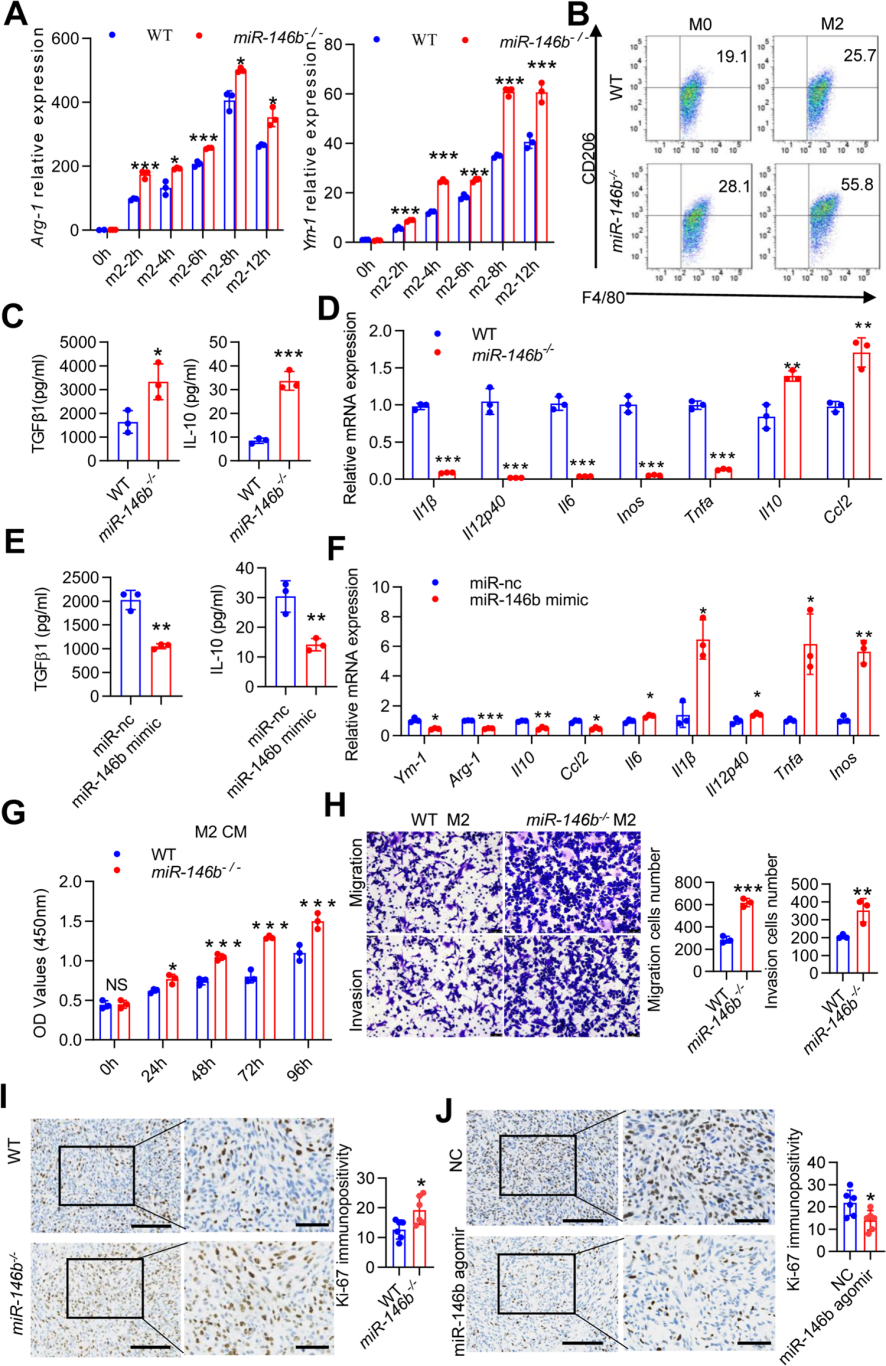
miR-146b mediates M2 macrophage differentiation. **(A)** BMDMs from WT or miR146b^−/−^ mice were stimulated with IL-4 (20 ng/ml) plus IL-10 (10 ng/ml) for various durations (0, 2, 4, 6, 8, 12 h), and *Arg-1* and *Ym-1* mRNA levels were determined by qPCR. **(B)** BMDMs from WT or miR146b^−/−^ mice were stimulated with IL-4 plus IL-10 for 24 h, stained for F4/80 and CD206, and analyzed by flow cytometry. Representative FACS dot plots gated on F4/80^+^ cells, and the percentage of CD206-positive cells are shown. **(C)** BMDMs were treated with IL-4 plus IL-10 for 24 h. The secreted IL-10 and TGFβ1 concentration in cell supernatants were determined by ELISA. **(D)** BMDMs were treated with IL-4 plus IL-10 for 12 h. *I1β*, *Il12p40*,* Il6*, *Inos*, *Tnfα*, *Il10* and *Ccl2* mRNA levels were determined by qPCR. **(E)** BMDMs originating from *miR-146b*^*−/−*^ mice were pretreated with miR-nc or miR-146b mimic for 12 h and then treated with IL-4 and IL-10 for 24 h. The secreted L-10 and TGFβ1 concentrations in cell supernatants were determined by ELISA. **(F)** BMDMs originating from *miR-146b*^*−/−*^ mice were pretreated with miR-nc or miR-146b mimic for 12 h and then treated with IL-4 and IL-10 for 12 h. *Arg-1* and *Ym-1* mRNA levels were determined by qPCR, and the secreted IL-10 and TGFβ1 levels were determined by ELISA. **(G)** MC38 cells were treated with conditioned medium originating from WT or *miR-146b*^*−/−*^ M2 cells for 0, 24, 48, 72 and 96 h. Cell viability was measured using the cell counting kit-8 (CCK-8) system. **(H)** M2 and MC38 cells were seeded into the lower and upper transwell chambers, respectively, and the coculture system was maintained for 24 h. Migrating and invading cells were fixed, stained, and counted. **(I)** Immunohistochemical staining to detect Ki-67 expression and semiquantitative score analysis of the excised tumor tissues from WT and *miR-146b*^*−/−*^ mice (*n* = 6). **(J)** Immunohistochemical staining to detect Ki-67 expression and semiquantitative score analysis of the excised tumor tissues from *miR-146b*^*−/−*^ mice after treatment with miR-nc or miR-146b mimic (*n* = 6). The data represent the mean ± SD. *p < 0.05; **p < 0.01; ***p < 0.001; NS, not significant

To determine whether miR-146b directly regulates macrophage polarization, we analyzed M2 markers and anti-inflammatory factor expression in murine BMDMs stimulated in vitro with anti-inflammatory (IL-4 plus IL10) conditions. The mRNA expression of M2 markers (*Arg-1* and *Ym-1*) was upregulated in miR-146b^−/−^ macrophages (Fig. 2A), and the percentages of CD206^+^F4/80^+^ cells were increased in miR-146b^−/−^ macrophages (Fig. 2B). These findings correlated with the enhanced secretion of IL-10 and TGFβ1 in miR-146b^−/−^ macrophages, as determined by ELISA (Fig. 2C). Quantitative analyses confirmed that genes associated with immune activation (*Il1β*, *Il12p40*, *Il6*, *Inos*, and *Tnfα*) were inhibited in miR-146b^−/−^ macrophages, and genes associated with immune suppression (*Il10* and *Ccl2*) were upregulated (Fig. 2D). Furthermore, the miR-146b mimic significantly suppressed the secretion of IL-10 and TGFβ1 and the expression of *Arg-1*, *Ym-1*, *Il10* and *Ccl2* but upregulated the expression of M1-related signature genes, such as* Il1β*, *Il12p40*, *Il6*, *Inos*, and *Tnfα* (Fig. 2E-F). These data confirmed that miR-146b modulated macrophage switching between immune stimulation and suppression.

TAMs in the TME maintain tumor growth and metastasis through the release of anti-inflammatory factors [6]. To investigate the effects of miR-146 on colon tumor cells in vitro, we conducted a series of experiments to elucidate the effect of miR-146b-deficient macrophages on MC38 cells. MC38 cells were stimulated with CM from WT or miR-146b^−/−^ macrophages, and Cell Counting Kit-8 (CCK8) assays were performed and showed that miR-146b^−/−^ CM significantly increased cell proliferation (Fig. 2G); CM derived from miR-146b^−/−^ macrophages also promoted MC38 cell migration and invasion (Fig. 2H). Referencing the xenograft tumor model shown in Fig. 1, the positive percentage of Ki67 was increased in MC38-bearing miR-146b^−/−^ mice (Fig. 2I) but was significantly decreased by miR-146b agomir treatment (Fig. 2J). These results further confirmed that miR-146b^−/−^ macrophages promoted CRC cell motility.

### Deletion of miR-146b in macrophages impairs T cell infiltration and promotes the immunosuppressive TME

**Fig. 3:**
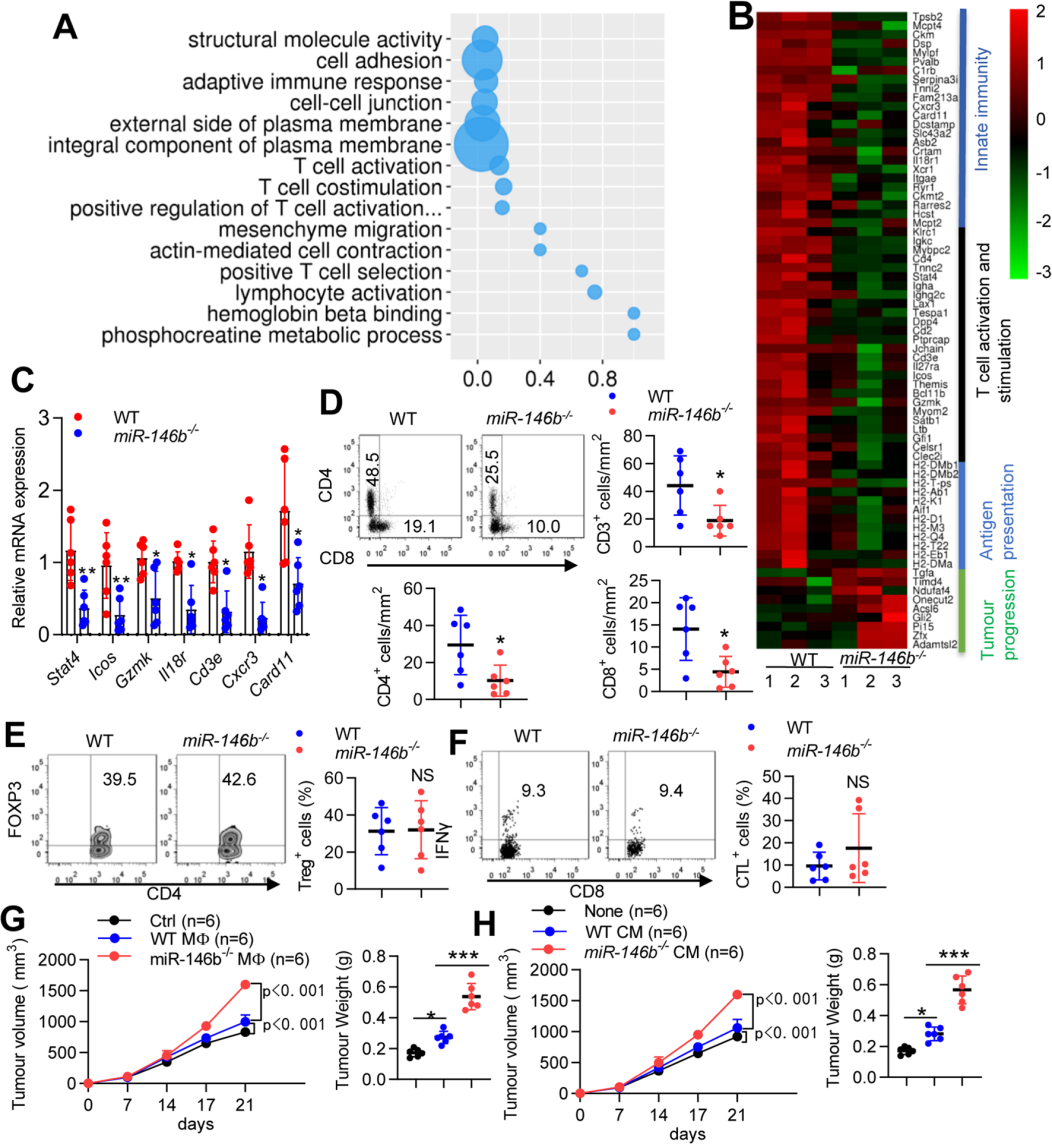
miR-146b deletion promotes T-cell exhaustion. **(A)** GO analysis of differentially expressed genes between tumors from WT and *miR-146b*^*−/−*^ mice. **(B)** Heatmap of differentially expressed immune response-related genes between tumors from WT and *miR-146b*^*−/−*^ mice. **(C)** Relative mRNA expression of immune response-related genes between tumors from WT and *miR-146b*^*−/−*^ mice. **(D)** Flow cytometric analysis and quantification of T-cell populations in tumors from WT and *miR-146b*^*−/−*^ mice. **(E–F)** Flow cytometric analysis and quantification of Tregs and CTLs in tumors from WT and *miR-146b*^*−/−*^ mice. **(G)** WT mice were implanted with MC38 cells mixed with in vitro cultured M2 macrophages (*n* = 6). Tumor growth and weight were monitored. **(H)** WT mice were implanted with MC38 cells in M2 macrophage-conditioned medium (CM) (*n* = 6). The data represent the mean ± SD. *p < 0.05; **p < 0.01; ***p < 0.001; NS, not significant

To understand how the TME was changed by miR-146b depletion, we performed RNA-seq and compared WT and *miR-146b*^*−/−*^ tumor-bearing mice. Transcriptome analyses of whole tumor tissue mRNA expression showed 111 upregulated and 138 downregulated genes in *miR-146b*^*−/−*^ mice compared with WT mice (Supplementary Fig. 2). As expected, Gene Ontology (GO) enrichment analysis indicated that a portion of the genes were associated with T cell activation and stimulation (Fig. 3A). The downregulated genes were enriched for molecules involved in “Innate immunity, T cell activation and stimulation and antigen presentation”, and the upregulated genes were enriched for molecules involved in “tumor progression” (Fig. 3B). These results were validated by qPCR on a second set of samples, including *Stat4*, *Icos*, *Gzmk*, *Il18r*, *Cd3e*, *Cxcr3* and *Card11* (Fig. 3C). Using flow cytometry (FACS), we further confirmed that the depletion of miR-146b decreased the infiltration of CD3^+^ T, CD4^+^ T and CD8^+^ T cells in tumors (Fig. 3D and Supplementary Fig. 1E), although Treg and CTLs did not significantly change (Fig. 3E-F).

Since miR-146b depletion inhibited proinflammatory responses in macrophages, we next examined whether macrophage miR-146b depletion slowed adaptive immunity and promoted tumor progression. BMDMs from WT and *miR-146b*^*−/−*^ mice were stimulated with the classic M2-polarizing cytokine IL-4 plus IL-10, mixed with MC38 cells and adoptively transferred into new WT recipient mice. Tumor growth was significantly promoted in the presence of M2 macrophages, and *miR-146b*^*−/−*^ M2 macrophages were significantly more likely to promote tumor progression than WT M2 macrophages (Fig. 3G). T cells were significantly decreased in tumors treated with *miR-146b*^*−/−*^ M2 cells compared with tumors treated with WT M2 macrophages (Supplementary Fig. 3A), confirming that miR-146b depletion in macrophages inhibited T cell recruitment to tumors. Moreover, the levels of proinflammatory factors in tumors treated with *miR-146b*^*−/−*^ M2 cells were significantly decreased, and anti-inflammatory factors were increased (Supplementary Fig. 3B). To determine whether macrophage-derived cytokines control tumor growth, we implanted MC38 cells mixed with CM derived from in vitro cultured macrophages into new WT mice. Tumor growth was enhanced by IL-4 plus IL-10-stimulated macrophage CM, and tumor growth was more obvious in cells mixed with CM derived from *miR-146b*^*−/−*^ macrophages (Fig. 3H). These results indicated that miR-146b deletion in macrophages impaired T cell infiltration in CRC tissues.

### miR-146b targets p110β and inhibits the PI3K/AKT signaling pathway in the regulation of M2 macrophage polarization

**Fig. 4:**
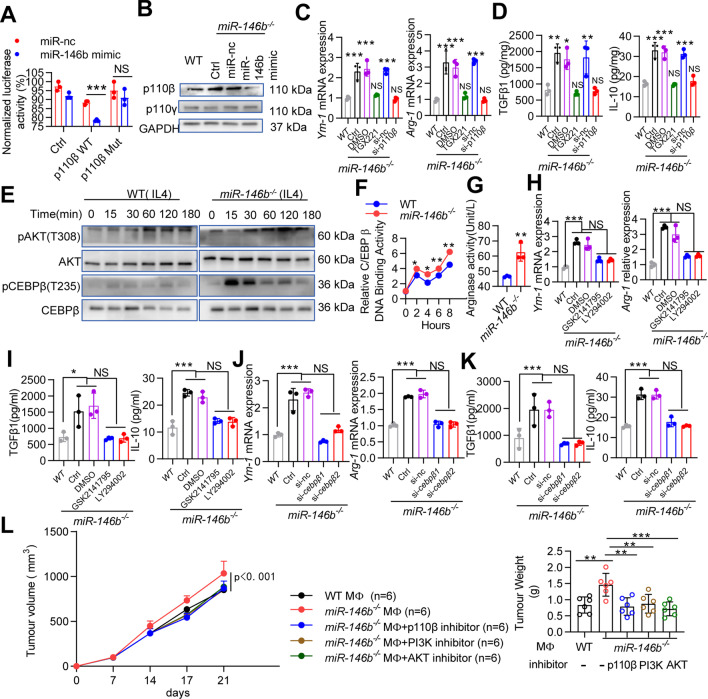
miR-146b regulates p110β/AKT/CEBPβ during M2 macrophage polarization. **(A)** 293 T cells were cotransfected with either WT or Mut p110β 3′UTR luciferase reporter plasmids together with miR-146b mimic for 24 h. The cell lysates were harvested, and luciferase activity was detected. **(B)** *miR-146b*^*−/−*^ BMDMs were pretreated with miR-nc or miR-146b mimic. Then, BMDMs were treated with IL-4 and IL-10 for 24 h. Western blots were used to detect p110β, p110γ and GAPDH protein levels. **(C-D)** *miR-146b*^*−/−*^ BMDMs were pretreated with si-*p110β* or inhibitors of p110β (TGX221) and then treated with IL-4 and IL-10. *Arg-1* and *Ym-1* mRNA levels were determined by qPCR. TGFβ1 and IL-10 protein levels were determined by ELISA. **(E)** Immunoblotting for pAkt/Akt and pC/EBPβ/C/EBPβ in IL-4 (20 ng/ml) stimulated WT and *miR-146b*^*−/−*^ BMDMs. **(F)** WT or *miR-146b*^*−/−*^ BMDMs were treated with IL-4 for 0–8 h, and C/EBPβ DNA-binding activity was assessed using the C/EBPβ Transcription Factor Assay Kit. **(G)** WT or *miR-146b*^*−/−*^ BMDMs were treated with IL-4 plus IL-10 for 24 h, and Arg-1 activity was determined. **(H-I)** *miR-146b*^*−/−*^ BMDMs were preincubated with inhibitors of AKT (GSK2141795) and PI3K (LY294002) for 30 min before the addition of IL-4 plus IL-10. *Arg-1* and *Ym-1* mRNA levels were determined by qPCR. TGFβ1 and IL-10 protein levels were determined by ELISA. **(J-K)** *miR-146b*^*−/−*^ BMDMs were pretreated with si-*cebpβ* for 12 h, and then treated with IL-4 and IL-10. *Arg-1* and *Ym-1* mRNA levels were determined by qPCR. TGFβ1 and IL-10 protein levels were determined by ELISA. **(L)** MC38 cells were mixed with M2 macrophages, and *miR-146b*^*−/−*^ M2 macrophages were pretreated with the p110β inhibitor (TGX221), the AKT inhibitor (GSK2141795) or the PI3K inhibitor (LY294002). Tumor growth and weight were monitored. All experiments were repeated three times independently. The data represent the mean ± SD. *p < 0.05; **p < 0.01; ***p < 0.001; NS, not significant

We used two publicly available algorithms to identify potential miR-146b target genes, and bioinformatics analysis showed that murine p110β was one of the predicted targets of miR-146b (data not shown), which is an isoform of PI3K that cooperates with p110γ to induce PI3K/AKT signaling activation [15]. We thus constructed reporter genes containing the p110β 3′ untranslated region (UTR) (Supplementary Fig. 4). We found that the miR-146b mimic significantly decreased the luciferase activity in 293 T cells transfected with the WT p110β plasmid; however, the miR-146b mimic had no effect on the mutant p110β plasmid (Fig. 4A). Further experiments showed that p110β protein levels were markedly increased in *miR-146b*^*−/−*^ M2 macrophages compared with WT M2 macrophages but dramatically decreased in cells with the miR-146b mimic (Fig. 4B). To confirm the effect of miR-146b on p110β during M2 polarization, *miR-146b*^*−/−*^ BMDMs were transfected with si-*p110β* or treated with a p110β inhibitor (TGX221) [16]. Knocking down or inhibiting p110β reduced *Arg-1* and *Ym-1* mRNA levels and cytokine secretion in *miR-146b*^*−/−*^ macrophages (Fig. 4C-D and Supplementary Fig. 5A). These results indicate that p110β is involved in the M2 differentiation of *miR-146b*^*−/−*^ macrophages.

AKT signaling is regulated by p110β [15], and the PI3K/AKT signaling pathway can control the switch between immune stimulation and suppression in cancer by the C/CATT enhancer binding protein (C/EBPβ) [8]. We examined whether miR-146b affects PI3K/AKT signaling and C/EBPβ phosphorylation and found that *miR-146b* deletion increased AKT and C/EBPβ phosphorylation (Fig. 4E). This result was confirmed by C/EBPβ transcription factor assay kits, the data showed that miR-146b ablation enhanced the DNA binding activity of C/EBPβ (Fig. 4F). C/EBPβ induces immune suppression by controlling Arg-1 expression [12, 17]. We found that Arg-1 activity was enhanced in *miR-146b*^*−/−*^ M2 macrophages (Fig. 4G). These results suggest that miR-146b is an upstream inhibitor of AKT signaling and C/EBPβ activation. We further found that a PI3K inhibitor (LY294002) and an AKT inhibitor (GSK2141795) could substantially abolish the effects of miR-146b deletion on the anti-inflammatory effects of M2 macrophages (Fig. 4H-I). In addition, *miR-146b*^*−/−*^ M2 macrophages treated with si-*Cebpβ* substantially abrogated the miR-146b deletion induced anti-inflammatory effect (Fig. 4J-K and Supplementary Fig. 5B).

To confirm that miR-146b controls tumor growth through the p110β-dependent PI3K/AKT pathway, we treated *miR-146b*^*−/−*^ macrophages ex vivo with inhibitors of p110β, PI3K and AKT prior to mixing the macrophages with tumor cells and implanting them in mice. Blockade of the p110β/PI3K/AKT pathway in *miR-146b*^*−/−*^ macrophages suppressed tumor growth (Fig. 4L). p110β/PI3K/AKT inhibition stimulated T cell recruitment (Supplementary Fig. 6A), enhanced the expression of proinflammatory factors and inhibited the expression of immunosuppressive factors (Supplementary Fig. 6B). These results indicate that miR-146b inhibits tumor growth through p110β/PI3K/AKT-mediated immune activation and that p110β/PI3K/AKT inhibition reverses these effects by polarizing macrophages toward a proinflammatory phenotype.

### PD-L1 is a critical downstream target of miR-146b in TAMs

**Fig. 5:**
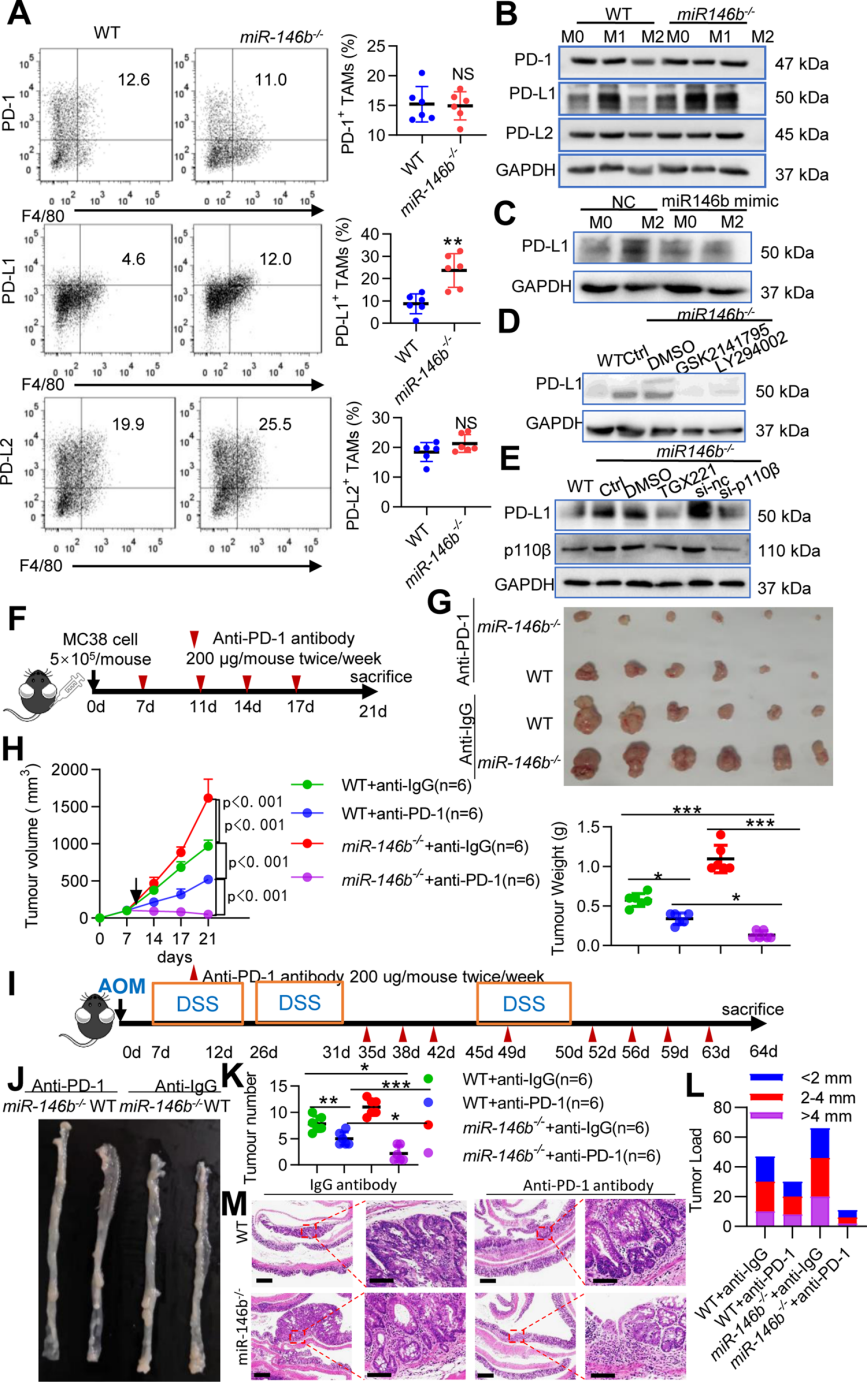
Anti-PD-1 antibody produces stronger effects in miR-146b^−/−^ mice. **(A)** Flow cytometry analysis of CD11b^+^Gr1^−^F4/80^+^CD206^+^PD1^+^, CD11b^+^Gr1^−^F4/80^+^CD206^+^PD-L1^+^ and CD11b^+^Gr1^−^F4/80^+^CD206^+^PD-L2^+^ cells in tumors from WT and *miR-146b*^*−/−*^ mice (*n* = 6). **(B)** BMDMs originating from WT and *miR-146b*^*−/−*^ mice were not treated or treated with IL-4 (20 ng/ml) plus IL-10 (10 ng/ml) or IFN-g (10 ng/ml) plus LPS (200 ng/ml) for 24 h. Cell lysates were prepared, and western blotting was performed to analyze PD-1, PD-L1 and PD-L2 protein expression. **(C)** BMDMs from *miR-146b*^*−/−*^ mice were pretreated with miR-nc or miR-146b mimic for 12 h and then treated with IL-4 plus IL-10 for 24 h. Cell lysates were prepared, and western blotting was performed to analyze PD-L1 protein expression. **(D)** BMDMs from WT or *miR-146b*^*−/−*^ mice were preincubated with an AKT inhibitor (GSK2141795) or a PI3K inhibitor (LY294002) for 30 min and then treated with IL-4 plus IL-10 for 24 h. Whole cell lysates were prepared, and western blotting was performed to analyze PD-L1 expression. **(E)** BMDMs from WT or *miR-146b*^*−/−*^ mice were pretreated with p110β inhibitor (TGX221) for 30 min or si-*cebpβ* for 12 h followed by treatment with IL-4 plus IL-10 for 24 h. Whole cell lysates were prepared, and western blotting was performed to analyze PD-L1 expression. **(F)** Schematic overview of the administration of anti-PD-1 antibodies in the MC38 model (*n* = 6). Mice were treated with anti-PD-1 antibodies (250 μg/mouse) or control IgG twice/week for 2 weeks. **(G)** Representative photograph showing tumor formation. **(H)** Tumor growth and weight were monitored. **(I)** Schematic overview of the administration of anti-PD-1 antibodies in the AOM-DSS model (*n* = 6). Mice were treated with anti-PD-1 antibodies (250 μg/mouse) or control IgG twice/week after the second DSS cycle. **(J)** Representative photograph showing tumor formation. **(K-L)** The number of colonic tumors and the tumor size distribution according to tumor number (tumor load) were recorded. **(M)** Representative HE-stained sections of colonic tumors (left panel scale bar, 200 μm; right panel scale bar, 100 μm). GAPDH expression served as a control. The data represent the mean ± SD. *p < 0.05; **p < 0.01; ***p < 0.001; NS, not significant

Since PD-L1 expression can be upregulated by activation of the AKT pathway [18], we hypothesized that miR-146b deletion might affect the expression of PD-L1 through p110β/PI3K/AKT signaling, and this upregulation indicates reinforced immune checkpoint blockade (ICB) efficacy [19]. Indeed, PD-L1 expression was increased in M2-TAMs from *miR-146b*^*−/−*^ tumor-bearing mice compared with those from WT mice, but PD-1 and PD-L2 expression was not significantly changed (Fig. 5A). We further tested PD-1, PD-L1 and PD-L2 protein expression in the M0, M1 and M2 states, and the protein expression of PD-L1 was higher in *miR-146b*^*−/−*^ M2 macrophages than in WT M2 macrophages (Fig. 5B). Moreover, the miR-146b mimic significantly suppressed the expression of PD-L1 in *miR-146b*^*−/−*^ M2 macrophages (Fig. 5C). To determine whether miR-146b influences PD-L1 expression through p110β/PI3K/AKT signaling in TAMs, we knocked down the p110β/PI3K/AKT pathway by siRNA or inhibitors, and genetic or pharmacological inhibition of p110β/PI3K/AKT signaling reduced PD-L1 expression in *miR-146b*^*−/−*^ M2 macrophages (Fig. 5D-E). These results indicate that miR-146b deletion promotes PD-L1 expression through p110β/PI3K/AKT signaling in M2 macrophages.

TAMs highly expressing PD-L1 are a predictive marker responsible for immunotherapy [2]. We found that the deletion of miR-146b upregulated PD-L1 expression on TAMs. This finding strongly suggests that treating *miR-146b*^*−/−*^ mice with anti-PD-1 therapy can result in an additional antitumor effect. In fact, in the murine MC38 colon cancer model (Fig. 5F), the administration of anti-PD-1 antibodies to *miR-146b*^*−/−*^ mice significantly delayed tumor growth compared to the administration of anti-PD-1 mAbs to WT mice (Fig. 5G-H). In addition, these findings were confirmed in another murine AOM/DSS model (Fig. 5I), and the numbers and sizes of macroscopically visible tumors from anti-PD-1 mAb-treated *miR-146b*^*−/−*^ mice were significantly decreased compared to those from anti-PD-1 mAb-treated WT mice (Fig. 5J-M). Collectively, these data suggested that miR-146b deletion enhanced PD-L1 expression in TAMs, thereby boosting ICB therapy.

### METTL3-dependent m^*6*^A methylation regulates the maturation of pri-miR-146b in macrophages

**Fig. 6:**
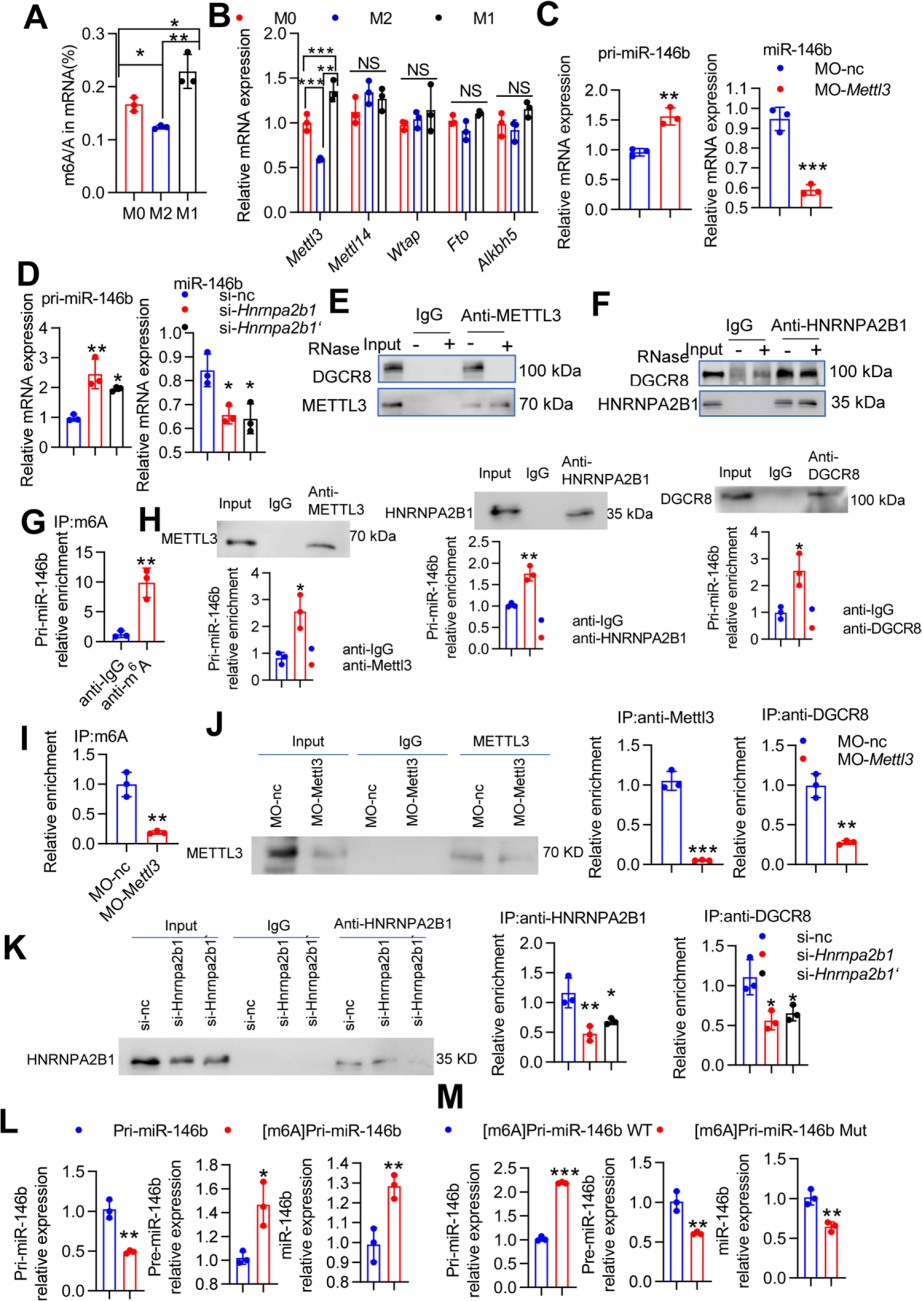
m^6^A modification mediates pri-miR-146b maturation in M2 macrophages. **(A-B)** BMDMs from WT mice were stimulated with IL-4 plus IL-10 (M2) or LPS plus IFNγ (M1), and the m^6^A levels of mRNAs were assessed using the EpiQuik m6A RNA Methylation Quantification Kit. *Mettl3*, *Mettl14*, *Wtap*, *Alkbh5* and *Fto* mRNA levels were determined by qPCR. **(C-D)** BMDMs from WT mice were transfected with MO-*Mettl3* or si-*hnrnpa2b1* for 24 h and then stimulated with IL-4 plus IL-10 for 4 h. The mRNA levels of *pri-miR-146b* and *miR-146b* were determined by qPCR. **(E)** RAW264.7 cells were treated with IL-4 (20 ng/ml) plus IL-10 (10 ng/ml) for 24 h to generate M2 macrophages. Coimmunoprecipitation of the METTL3-interacting protein DGCR8 was performed. After immunoprecipitation, the samples were washed and incubated with RNase as indicated. Western blotting was used to detect METTL3 and DGCR8, and IgG served as a control for immunoprecipitation. **(F)** RAW264.7 cells were treated with IL-4 plus IL-10 for 24 h. Coimmunoprecipitation of the HNRNPA2B1-interacting protein DGCR8 was performed. After immunoprecipitation, the samples were washed and incubated with RNase as indicated. Western blotting was used to detect HNRNPA2B1 and DGCR8. **(G)** RAW264.7 cells were treated with IL-4 plus IL-10 for 24 h. Coimmunoprecipitation was performed with an anti-m^6^A antibody. After immunoprecipitation, the samples were washed, and pri-miR146b expression was determined by qRT-PCR. **(H)** RAW264.7 cells were treated with IL-4 plus IL-10 for 24 h. Western blot analysis was used to detect METTL3, HNRNPA2B1 and DGCR8 expression in the input or immunoprecipitation group, and RIP assays showed significant enrichment of *pri-miR146b* in RNA binding to METTL3, HNRNPA2B1 and DGCR8. **(I)** RAW264.7 cells were transfected with MO-*Mettl3* for 24 h and then stimulated with IL-4 plus IL-10 for 4 h. Immunoprecipitation of m^6^A-modified RNA in control or METTL3-knockdown cells was followed by qRT-PCR to assess pri-miR146b m^6^A modification levels. **(J)** Western blot analysis was used to detect METTL3 expression in the input and immunoprecipitation group, and RIP assays showed pri-miR146b binding to METTL3 or DGCR8. **(K)** RAW264.7 cells were transfected with si-*hnrnpa2b1* for 24 h and then stimulated with IL-4 plus IL-10 for 4 h. Western blot analysis was used to detect HNRNPA2B1 expression in the input and immunoprecipitation group, and RIP assays showed pri-miR146b binding to HNRNPA2B1 or DGCR8. **(L)** In vitro reaction system containing the starting materials of pri-miR-146 or [m^6^A]pri-miR-146 and the whole cellular lysates of 293 T cells transfected with plasmids carrying DROSHA and DGCR8. *pri-miR-146b*, *pre-miR-146b* and *miR-146b* mRNA levels were determined by qPCR. Pri-miR-1–1, which has no methylation site, was included as a control. **(M)** In vitro reaction system containing the starting materials of [m^6^A]pri-miR-146 WT or [m^6^A]pri-miR-146 Mut. *pri-miR-146b*, *pre-miR-146b* and *miR-146b* mRNA levels were determined by qPCR. All experiments were repeated three times independently. The data represent the mean ± SD. *p < 0.05; **p < 0.01; ***p < 0.001; NS, not significant

Recent studies have reported that m^6^A modification is involved in the life cycle of RNA and in primary microRNA (pri-miRNA) processing [20]. However, the potential roles of m^6^A modification in miR-146b transcription and maturation remain elusive. To study the effects of m^6^A modification on the processing of pri-miRNA-146b to mature miR-146b in the context of macrophage differentiation, we first examined whether m^6^A levels varied with macrophage differentiation under polarized conditions. The m^6^A RNA methylation level was analyzed in BMDMs under resting macrophage (M0), M1 and M2 type macrophage conditions. The results showed that M2-type stimulation inhibited the m^6^A modification compared to that in the M0 and M1 states (Fig. 6A). The m^6^A-related gene *Mettl3* was notably decreased under M2 type stimulation, but levels of the methyltransferase genes *Mettl14* and *Wtap* as well as the demethylase genes *Alkbh5* and *Fto* did not change significantly (Fig. 6B). To investigate the biological relevance of the m^6^A modification in macrophage differentiation, we used MO-*Mettl3* [21], which decreased METTL3 protein levels (Supplementary Fig. 7A). We found that pri-miR-146b expression was significantly increased, but mature miR-146b expression was downregulated by MO-*Mettl3* compared to that in the control group (Fig. 6C). The m^6^A “reader” protein HNRNPA2B1 binds to m^6^A in pri-miRNA transcripts to promote primary miRNA processing by interacting with the microRNA microprocessor complex protein DGCR8 [22]. HNRNPA2B1 knockdown caused pri-miR-146b upregulation and a decline in mature miR-146b (Supplementary Fig. 7B and Fig. 6D). Using a coimmunoprecipitation assay, we also confirmed that the METTL3 interaction with DGCR8 was mediated by RNA [23], given that RNase treatment disrupted this association (Fig. 6E). In addition, HNRNPA2B1 interacted with DGCR8 through protein–protein interactions (Fig. 6F) [22].

To demonstrate the direct role of the m^6^A modification in pri-miR-146b in mature miR-146b processing, m^6^A-specific RNA immunoprecipitation (RIP) coupled with qRT-PCR was used, and the results showed that pri-miR-146b was significantly enriched by the anti-m^6^A antibody (Fig. 6G). Subsequent RIP-qPCR analysis showed that the METTL3, HNRNPA2B1 and DGCR8 proteins interacted with pri-miR-146b (Fig. 6H). Furthermore, we found that the deceased level of pri-miR-146b was immunoprecipitated by anti-m^6^A in METTL3-knockdown cells and was immunoprecipitated by anti-DGCR8 and anti-METTL3 antibodies (Fig. 6I-J). In addition to METTL3 silencing, HNRNPA2B1 knockdown substantially reduced the interaction of HNRNPA2B1 and DGCR8 with pri-miR-146b (Fig. 6K). These data suggested that METTL3, DGCR8 and HNRNPA2B1 could disturb the interaction between m^6^A and pri-miR-146b. To directly confirm that the processing of pri-miR-146b to mature miR-146b was mediated by m^6^A modification, we performed an in vitro pri-miRNA processing assay to examine this relationship. We observed that [m^6^A] pri-miR-146b was more rapidly and efficiently processed to pre-miR-146b followed by mature miR-146b than its unmethylated counterpart (Fig. 6L, Supplementary Fig. 8). However, when the potential m^6^A motif in pri-miR-146b was mutated, the processing of pri-miR-146b to mature miR-146b was inhibited (Fig. 6M). Taken together, these results suggest that METTL3 and HNRNPA2B1 promote the processing of pri-miR-146b to mature miR-146b in an m^6^A-dependent manner.

### METTL3 modulates TAMs via the miR-146b/p110β/PI3K/AKT axis

**Fig. 7:**
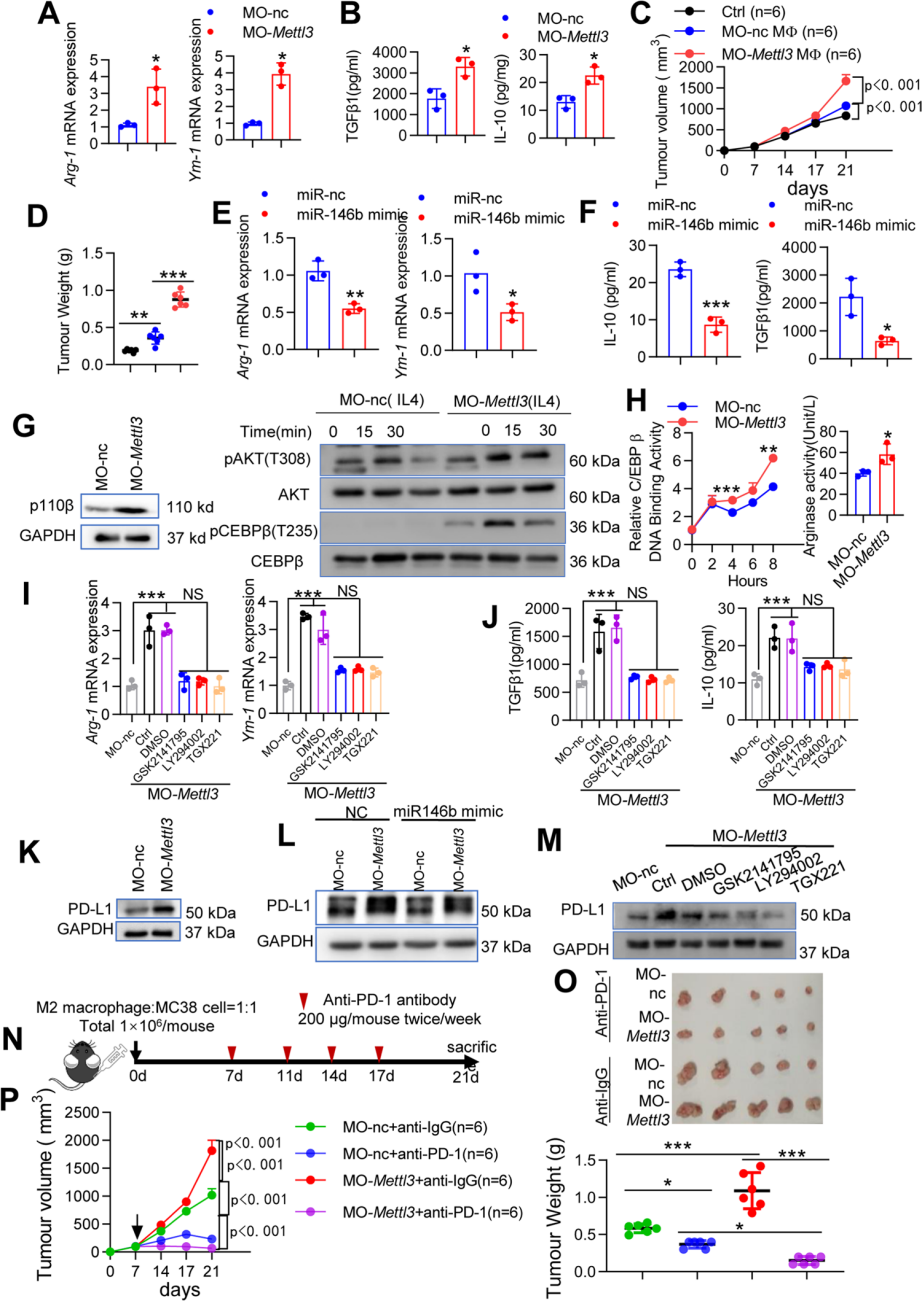
METTL3 mediates TAM polarization and PD-L1 expression via the miR-146b/p110β/PI3K/AKT axis in vitro and in vivo. **(A)** BMDMs from WT mice were transfected with MO-*Mettl3* for 24 h and then stimulated with IL-4 plus IL-10 for 4 h. *Arg-1* and *Ym-1* mRNA levels were determined by qPCR. **(B)** BMDMs from WT mice were transfected with MO-*Mettl3* for 24 h and then stimulated with IL-4 plus IL-10 for 24 h. TGFβ1 and IL-10 protein levels were determined by ELISA. **(C-D)** BMDMs from WT mice were transfected with MO and then treated with IL-4 plus IL-10. WT mice were implanted with MC38 cells mixed with in vitro cultured M2 macrophages (*n* = 6). Tumor growth and weight were monitored. **(E–F)** BMDMs from WT mice were transfected with MO-*Mettl3* for 12 h, transfected with miR-146b mimic for another 12 h, and then stimulated with IL-4 plus IL-10 for 4 h or 24 h. The mRNA levels of Arg-1 and Ym-1 were determined by qPCR. TGFβ1 and IL-10 protein levels were determined by ELISA. **(G)** BMDMs from WT mice were transfected with MO-*Mettl3* for 12 h and then stimulated with IL-4 plus IL-10 for the indicated times. The expression of p110β, pAkt/Akt, pC/EBPβ, and C/EBPβ was determined by western blotting. **(H)** BMDMs from WT mice were transfected with MO-*Mettl3* for 12 h, and then stimulated with IL-4 plus IL-10. C/EBPβ DNA-binding activity and Arg-1 activity were determined. **(I-J)** BMDMs from WT mice were transfected with MO-*Mettl3* for 12 h, preincubated with an AKT inhibitor (GSK2141795) or a PI3K inhibitor (LY294002) for an additional 30 min and then treated with IL-4 plus IL-10. *Arg-1* and *Ym-1* mRNA levels were determined by qPCR. TGFβ1 and IL-10 protein levels were determined by ELISA. **(K)** BMDMs from WT mice were transfected with MO-*Mettl3* for 12 h and then stimulated with IL-4 plus IL-10. PD-L1 expression was determined by western blotting. **(L)** BMDMs from WT mice were transfected with MO-*Mettl3* for 12 h, transfected with miR-146b mimic for an additional 12 h, and then stimulated with IL-4 plus IL-10. PD-L1 expression was determined by western blotting. **(M)** BMDMs from WT mice were transfected with MO-*Mettl3* for 12 h; preincubated with GSK2141795, LY294002 or TGX221 for an additional 30 min and then treated with IL-4 plus IL-10. PD-L1 expression was determined by western blotting. **(N)** Schematic overview of the administration of anti-PD-1 antibodies in the MC38 model (*n* = 6). Mice were treated with anti-PD-1 antibodies (250 μg/mouse) or control IgG twice/week for 2 weeks. **(O)** Representative photograph showing tumor formation. **(P)** Tumor growth and weight were monitored. All experiments were repeated three times independently. The data represent the mean ± SD. *p < 0.05; **p < 0.01; ***p < 0.001; NS, not significant

m^6^A deposition influences CRC growth [24, 25], and the protumoral function of TAMs is promoted by reduced m^6^A levels [26]. To determine METTL3 function in M2 macrophage differentiation, we assessed macrophage development after METTL3 knockdown. As expected, the M2-TAMs markers *Arg-1* and *Ym-1* were significantly increased in MO-*Mettl3*-treated macrophages compared to MO–nc cells (Fig. 7A). ELISA analysis showed that the secretion of IL-10 and TGF-β1 was significantly increased in MO-*Mettl3*-treated macrophages (Fig. 7B). To further confirm that macrophage METTL3 controls tumor growth, BMDMs derived from WT mice were transfected with MO–nc or MO-mettl3, mixed with MC38 cells, and then adoptively transferred into new WT recipient mice. Knocking down *Mettl3* in macrophages promoted MC38 tumor growth (Fig. 7C-D). Consistently, impaired T cell infiltration into MC38 cell-derived tumor tissues occurred when the cells were mixed with MO-*Mettl3* macrophages compared with cells mixed with MO–nc (Supplementary Fig. 9A). Accordingly, TGF-β1, IL-10 and CCL2 protein levels were significantly increased, and TNFα, IL-6, IL-1β and IL-12p40 protein levels were significantly decreased in tumor tissues in mice with MO-*Mettl3* macrophages compared with mice with MO–nc macrophages (Supplementary Fig. 9B).

To further confirm the role of miR-146b as a downstream target of METTL3 in the polarization of TAMs, the miR-146b mimic was administered to METTL3-knockdown cells. *Arg-1* and *Ym-1* mRNA and IL-10 and TGFβ1 protein secretion levels were decreased (Fig. 7E-F). Moreover, we assessed the p110β/PI3K/AKT axis. As expected, METTL3 knockdown significantly increased p110β expression and AKT and C/EBPβ phosphorylation (Fig. 7G). C/EBPβ transcription and Arg-1 activity results also confirmed that METTL3 mediates the p110β/PI3K/AKT axis in M2 macrophages (Fig. 7H). In addition, we silenced the p110β/PI3K/AKT pathway through the use of inhibitors in METTL3-knockdown M2 macrophages and found that inhibition of p110β/PI3K/AKT signaling significantly reversed the expression of *Arg1*, *Ym-1*, IL-10, and TGF-β1 induced by METTL3 knockdown (Fig. 7I-J). These results suggesting that the miR-146b/p110β/PI3K/AKT axis at least partially mediates METTL3 function in TAM differentiation.

### Loss of METTL3 sensitizes CRC to anti-PD-1 treatment

We next examined the role of METTL3 in PD-L1 expression in M2 macrophages. In addition to the effect of miR-146b, PD-L1 protein expression was significantly elevated in the METTL3-knockdown M2 macrophages (Fig. 7K) but reduced in the METTL3-knockdown M2 macrophages upon treatment with miR-146b mimic (Fig. 7L). In addition, PD-L1 protein levels were obviously decreased in the METTL3-knockdown M2 macrophages upon treatment with GSK2141795, LY294002 or TGX221 (Fig. 7M).

A murine MC38 colon cancer model was employed to investigate the effect of METTL3-knockdown on PD-1 blockade therapy. M2 macrophages with METTL3-knockdown mixed with MC38 cells were subcutaneously injected into C57BL/6 mice, and mice were treated with anti-PD-1 antibody (Fig. 7N). METTL3-knockdown M2 macrophages promoted tumor growth in tumor-bearing C57BL/6 mice. The administration of anti-PD-1 antibody to mice with treated with METTL3-knockdown M2 macrophages led to a more significant delay in tumor growth compared to those receiving anti-PD-1 antibody with control M2 macrophages (Fig. 7O-P). Together, our findings indicate that m^6^A methylation is a critical epigenetic modification for TAM differentiation in colorectal cancer. Moreover, METTL3 knockdown promotes CRC aggressiveness through downregulation of miR-146b.

## Discussion

In this study, we demonstrate that the METTL3/miR-146b axis reprograms macrophages to exert protumor effects. METTL3 knockdown or miR-146b ablation alone significantly increases the TAM population, reduces T cell infiltration, and promotes tumor progression. However, METTL3 or miR-146b deficiency increases PD-L1 expression in TAMs, and the therapeutic effect of anti-PD1 mAb is markedly improved and leads to the regression of even well-established CRC tumors. Mechanistically, we demonstrated that miR-146b deficiency promoted the polarization of TAMs by inducing oncogenic PI3K-AKT signaling; specifically, miR-146b expression is decreased in TAMs, which significantly increases p110β expression and promotes PI3K/AKT signaling activation. Moreover, we found that the m^6^A modification was the main factor associated with miR-146b expression and determined that knockdown of the m6A “writer” protein METTL3 and the “reader” protein HNRNPA2B1 significantly decreased miR-146b expression and promoted M2 macrophage polarization. These data suggest that the METTL3/miR-146b axis may represent an effective therapeutic target to reprogram TAMs, improve the immunosuppressive microenvironment of CRC tumors, and increase the efficacy of immunotherapy.

Many innate and adaptive immune cells are recruited to the TME, and macrophages are the most abundant immune cells [27]. Macrophages are the first line of defense against pathogenic insults, suggesting that these cells can be tumoricidal after being activated; however, cancer-associated macrophages are usually polarized to a protumoral M2 phenotype at the time of tumor initiation, and these cells are named TAMs [28]. Therefore, the polarization of TAMs can indirectly stimulate CTL activation and synergize with checkpoint inhibitors, suggesting improved cancer outcomes [8]. MiRNAs have also been identified as important determinants of the protumoral activity of TAMs. In our study, we observed that miR-146b participated in the initiation and progression of CAC, which significantly reduced tumor numbers and tumor areas [13]. Accordingly, Arg-1 expressing macrophages were sharply reduced [13]. *miR-146b*^*−/−*^ mice had significantly increased M2-TAM numbers, reduced T cell recruitment, and promoted immune suppression and tumor progression. Our in vitro study further confirmed that the miR-146b mimic could inhibit M2 macrophage polarization under M2 conditions.p110γ is the most highly expressed PI3K isoform in myeloid cells, and PI3K/AKT is involved in the macrophage phenotype transitions in the TME [8, 12]. We found that miR-146b ablation promoted PI3K/AKT signaling pathway activation, exacerbated the upregulation of the PI3K/AKT signaling pathway-associated target C/EBPβ in M2 macrophages and modulated Arg-1 expression by binding to the response element of Arg-1. These results suggest that miR-146b repressed the polarization of TAMs by targeting the PI3K/AKT signaling pathway. In our study, miR-146b deficient BMDMs promoted p110β expression but not p110γ expression. Intriguingly, both p110β and p110γ contribute to AKT activation [15]. Taken together, these results suggest that miR-146b can regulate the polarization of TAMs through PI3K/AKT signaling and that these activities may be highly dependent on p110β expression.

TAMs can suppress T cell recruitment and activation, thereby exacerbating immunosuppression [8]. We found that miR-146b deficiency reduced the T cells recruited to tumors. In addition, PD-L1 expression can be mediated by PI3K/AKT signaling [18].These effects indicate that miR-146b may affect T cell-based immunotherapy. David L. Rimm et al. recently showed that macrophages expressing PD-L1 can enhance anti-PD-1 mAb therapy [10]. We assessed the percentage of PD-L1 in TAMs and showed that PD-L1 expression in TAMs was significantly increased in macrophage bearing tumors. Furthermore, we found that high expression of PD-L1 in *miR-146b*^*−/−*^ macrophages indicated a better immunotherapeutic effect. Given that these outcomes occurred in *miR-146b*^*−/−*^ mice, we hypothesize that miR-146b deficiency-mediated polarization of TAMs promotes tumor progression, whereas increased PD-L1 expression potentially drives a response to anti-PD1 immunotherapy.

RNA m^6^A modification is a common epigenetic regulation that is involved in a variety of cellular processes [29, 30]. The decrease in the m^6^A “reader” METTL14 in TAMs determines T cell phenotypes in tumors [26]. In our study, m^6^A modification was decreased in M2 macrophages by the m^6^A methyltransferase METTL3, and we found that m^6^A methylation mediated the maturation process of pri-miR-146b. Knockdown of both METTL3 and the m^6^A “reader” HNRNPA2B1 impaired the maturation process of pri-miR-146b and led to a decrease in mature miR-146b expression. Furthermore, knockdown of both METTL3 and HNRNPA2B1 induced M2 macrophage polarization. When miR-146b mimics were transfected into METTL3-knockdown cells, the miR-146b mimic rescued the M2 macrophage polarization induced by METTL3. An in vivo study further confirmed that knockdown of METTL3 could decrease miR-146b expression, leading to an increase in TAMs and ultimately promoting tumor progression. More interestingly, we showed that METTL3 knockdown increased PD-L1 expression via miR-146b in M2 macrophages, thereby activating the p110β/PI3K/AKT pathway and inducing an immunosuppressive status. More interestingly, METTL3 knockdown led to sensitization of macrophages to anti-PD-1 therapy. These results suggested that METTL3 promotes the maturation of pri-miR-146b in CRC TAMs in an m^6^A-dependent manner. Targeting the enzymatic activity of METTL3 may be an anti-CRC strategy. For example, STM2457, the first bioavailable inhibitor of METTL3, may be useful, though future research is needed. In addition, we only quantified the m^6^A level in M2 macrophages using a colorimetric method, which was not quite accurate. Further might quantify m^6^A levels by mass spectrometry, and these results need to be confirmed in TAMs from human CRC tissues.

In conclusion, we have identified the role of the m^6^A-miR-146b/p110β/PI3K/AKT axis in the polarization of TAMs in CRC tissue, which might be associated with CRC development and progression. Our results also suggest that m^6^A modification may play an important role in CRC and other types of cancer linked with miR-146b decrease.

### Supplementary Information

Below is the link to the electronic supplementary material.

Supplementary file1 (DOCX 1161 KB)

## Data Availability

The data sets supporting the conclusions of this article are included within the article.

## Abbreviations

miR-146b: MicroRNA-146b
CRC: Colorectal cancer
TAMs: Tumor-associated macrophages
m^6^A: N6-methyladenosine
ICB: Immune checkpoint blockade
METTL3: Methyltransferase-like 3
PI3K: Phosphoinositide 3-kinase
PD-L1: Programmed death ligand 1
CTLA-4: Cytotoxic T lymphocyte-associated protein 4
PD-1: Programmed cell death 1
CTL: Cytotoxic CD8^+^ T cell
pMMR-MSI-L: Mismatch-repair-proficiency and microsatellite instability-low
CSF: Colony stimulating factor
TGF-β1: Transforming growth factor
IL: Interleukin
EGF: Epidermal growth factor
TME: Tumor microenvironment
miRNAs: MicroRNAs
CAC: Colitis-associated cancer
mAbs: Monoclonal antibodies
CLO: Clodronate
BMDMs: Bone marrow derived macrophages
AOM: Azoxymethane
DSS: Dextran sodium sulphate
CCK8: Cell Counting Kit-8
GO: Gene Ontology
FACS: Flow cytometry
MO: Morpholino
RIP: RNA immunoprecipitation
C/EBPβ: C/CATT enhancer binding protein
PBS: Phosphate-buffered saline
