# Supplementary material for: Modulation of miR-146b by N6-methyladenosine modification remodels tumor-associated macrophages and enhances anti-PD-1 therapy in colorectal cancer
Source: Cell Oncol (Dordr). 2023 Jul 5;46(6):1731–46. doi: 10.1007/s13402-023-00839-0 (PMC10697876; doi:10.1007/s13402-023-00839-0)
Supplement: Supplementary file 1 — Supplementary file1 (DOCX 1161 KB) [file 13402_2023_839_MOESM1_ESM.docx]

**Material and methods**

**Immunohistochemistry**

Deparaffinized colon sections were immunoassayed for Ki67 (Cell Signaling Technology, Beverly, MA, USA). The stained sections were examined with a Leica DM6 microscope (Leica, Wetzlar, Germany).

**Cell lines**

The mouse colon adenocarcinoma cell line MC38, mouse macrophage cell line RAW264.7 and HEK293T cell line were purchased from the Type Culture Collection of the Chinese Academy of Sciences (Shanghai, China). The cells were supplemented with 10% fetal bovine serum (FBS) and incubated with 5% CO_2_ at 37 ℃. To induce M2 macrophages, the RAW264.7 cell line was treated with IL-4 (20 ng/ml, PeproTech, Rocky Hill, NJ) plus IL-10 (10 ng/ml, PeproTech) for 24 h.

**Preparation of** **bone marrow-derived macrophages (BMDMs)**

Isolated bone marrow (BM) cells were used as previously reported^2^. The cells were cultured in 10% FBS medium supplemented with GM-CSF (10 ng/ml, PeproTech). On day 7, cells further treated with IL-4 (20 ng/ml, PeproTech) plus IL-10 (10 ng/ml, PeproTech) were considered M2 macrophages. For M1 macrophage polarization, on day 7, cells were further treated with 20 ng/ml interferon γ (IFNγ, PeproTech) plus 20 ng/ml lipopolysaccharide (LPS, PeproTech).

The negative control siRNA and mouse *cebpβ, p110β* and *hnrnpa2b1* siRNAs were obtained from Genepharma (Suzhou, China). The specific si-*cebpβ*, si-*p110β*, and si-*hnrnpa2b1* sequences were generated with the primers in Supplementary Table 1. The negative control and miR-146b mimic were ordered from Genepharma, and the sequences are presented in Supplementary Table 1. Vivo-morpholino negative control (MO-nc) and Vivo-morpholino oligos against mouse *Mettl3* (MO-*Mettl3*) were purchased from Gene Tools (Philomath, OR, USA), and the sequences are presented in Supplementary Table 1. BMDMs from *miR-146b^-/-^* mice were transfected with si-*p110β*, si-*cebpβ* or miR-146b mimic for 12 h, and the cells were then stimulated with IL-4 plus IL-10 for 4 h or 24 h. BMDMs from WT mice were transfected with si-*hnrnpa2b1* or MO for 12 h and then stimulated with IL-4 plus IL-10 for 4 h or 24 h. In some studies, *miR-146b^-/-^* BMDMs were preincubated with inhibitors of AKT (GSK2141795, 30 μM, Beyotime, Shanghai, China), PI3K (LY294002, 20 μM, Beyotime) and p110β (TGX221, 10 μM, Beyotime) for 30 min before IL-4 and IL-10 addition.

**Cell proliferation assay**

MC38 cells were seeded into 96-well plates at a density of 2000 cells per well and then treated with conditioned medium from WT or *miR-146b^-/-^* M2 cells (24 h of culture) for 0, 24, 48, 72 and 96 h. Cell viability was measured by a cell counting kit-8 (CCK-8) system (Dojindo, Japan) according to the manufacturer’s instructions.

**Cell migration and invasion assay**

The standard procedure was performed as previously described ^3^. Briefly, BMDMs were treated with IL-4 plus IL-10 for 24 h, and the medium was then changed. Macrophages (5×10^4^) and MC38 (5×10^4^) cells were seeded into the lower and upper Transwell chambers (24-well insert; 8 μm pore, Corning, USA), respectively. For the cell invasion assay, the upper chamber was coated with Matrigel (Corning). Migrated and invaded cells were fixed, stained, and counted in 5 random fields of view after 24 hours.

**Flow cytometry analysis**

For *in vitro* macrophage polarization, cells were stained with anti-mouse CD206 APC (eBioscience, San Diego, CA, USA) and anti-mouse F4/80-FITC (eBioscience) antibodies. For the immune cells isolated from the tumor tissue, cells were stained with anti-NK1.1-BV510 antibody. For macrophage analysis, anti-mouse CD11b-BV510 (BD Biosciences, San Jose, CA, USA), anti-mouse GR1-PE-Cy7 (eBioscience), anti-mouse F4/80-FITC, anti-mouse CD206-APC (eBioscience), anti-mouse MHC II-PE (eBioscience), anti-mouse PD-L2-BV421 (BD Biosciences), anti-mouse PD-L1-Percp-Cy5.5 (BD Biosciences) and anti-mouse PD-1-APC-Cy7 (BD Biosciences) antibodies were used. For intracellular staining, cells were stimulated with Cell Stimulation and Protein Transport Inhibitor Cocktail (eBioscience) for 4 h, stained with anti-mouse CD3-APC-Cy7 (BD Biosciences), anti-mouse CD4-FITC (eBioscience) and anti-mouse CD8-V450 (BD Biosciences) antibodies, and then stained using the Cytofix/Cytoperm Staining Kit (BD Biosciences) and anti-interferon gamma (IFNγ)-Percp-Cy5.5 (eBioscience) and anti-forkhead box protein 3 (FOXP3)-APC (eBioscience) antibodies. Flow cytometry was performed on a FACSVerse cytometer (BD Biosciences), and the results were subsequently analyzed using FlowJo 7.6 (Tree Star, Ashland, OR, USA).

**ELISA**

Cell supernatant concentrations of IL-10 and TGFβ1 were determined using an ELISA kit (4A Biotech, Beijing, China) according to the manufacturer’s instructions. Whole tumors were lysed in RIPA buffer, and the total protein concentration was determined using a BCA Protein Assay (Beyotime). Five hundred micrograms of total protein lysate from tumors were used in ELISAs to detect CCL2, TGFβ1, IL-1β, tumor necrosis factor α (TNFα), IL-6, IL-10 and IL-12p40 (4A Biotech). Protein expression was normalized to total volume (supernatants) or mg total protein (tumor lysates).

**Quantitative colorimetric arginase and transcription factor assays**

The QuantiChrom Arginase Assay Kit (DARG-200, BioAssay Systems, USA) was used to measure arginase activity in primary murine bone marrow derived macrophages from WT and *miR-146b^-/-^* mice according to the manufacturer’s instructions. To determine C/EBPβ activation, C/EBP Transcription Factor Assay Kits (Active Motif, Carlsbad, CA, USA) were used according to the manufacturer’s protocol^4^.

**Transfection and luciferase reporter assay**

293T cells were transfected with WT or Mut p110β 3`UTR luciferase reporter plasmid in the presence of miR-nc or miR-146b mimic. The plasmid was mixed with Lipofectamine 3000 (Invitrogen, Carlsbad, CA, USA) and then added to 12-well plates for 24 h. The cells were lysed (Beyotime) to determine luciferase activity.

**Quantitative real-time PCR**

Total RNA was extracted with TRIzol reagent (Thermo Fisher Scientific, Waltham, MA, USA), and cDNA was synthesized using PrimeScript^TM^ RT Master Mix (Takara, Dalian, China). Quantitative real-time PCR was performed with the primers in Supplementary Table 1 using SYBR Premix Ex Taq (Vazyme, Nanjing, China) and the Bio-Rad CFX96 System (Bio-Rad Laboratories, CA, USA). The pri-miR-146b, pre-miR-146b, miR-146b, pri-1-1 and U6 small nuclear RNAs were obtained from Qiagen (Hilden, Germany). The levels in samples were normalized to the levels of the housekeeping gene glyceraldehyde 3-phosphate dehydrogenase (*Gapdh*). For pre-miR-146b and miR-146b quantiﬁcation, U6 served as an internal control, and pri-miR-146b was normalized to *Gapdh*. The mRNA analysis was performed using the comparative 2^-△△CT^ method.

**Western blot analysis**

For western blot analyses, total protein samples were isolated by adding RIPA lysis buffer containing protease inhibitor cocktail tablets (Roche, Basel, Switzerland) and phosphatase inhibitor tablets (Roche) to tissue or cell samples. The primary antibodies used were as follows: anti-p110β, anti-p110γ, anti-phospho-AKT (T308), anti-AKT, anti-phospho-C/EBPβ (T235), and anti-C/EBPβ antibodies were purchased from Cell Signaling Technology, and anti-GAPDH (Zhonshanjinqiao, Wuhan, China). Anti-PD-1, anti-PD-L1 and anti-PD-L2 antibodies were purchased from Abcam (Cambridge, MA, USA). A Gene5 image acquisition system (Syngene, Frederick, MD, USA) was used for signal detection.

**RNA-seq**

Total RNA was extracted from tumor tissue. RNA-seq data were analyzed according to protocols that were previously described^5^. For RNA-seq analysis, genes were considered significantly differentially expressed if they showed a ≥2-fold change and < 0.01 *P* value. Enriched KEGG pathways were obtained and volcano plots were generated through online bioinformatics tools.

**m^6^A quantification**

Total RNA was extracted by using TRIzol. The EpiQuik m^6^A RNA Methylation Quantification Kit (Colorimetric) (Epigentek, NY, USA) was utilized to measure the global m^6^A levels in mRNA following the manufacturer’s protocol^6^.

**Coimmunoprecipitation assay**

Cells were lysed with LB1 (50 mM HEPES-KOH pH 7.5, 140 mM NaCl, 1 mM EDTA, 10% glycerol, 0.5% Triton X-100, and protease inhibitors) and diluted 10-fold in dilution buffer (50 mM Tris-HCl pH 7.4, 100 mM NaCl). Immunoprecipitation was performed with anti-HNRNPA2B1 rabbit antibody (Abcam) and anti-METTL3 rabbit antibody (Abcam) bound to Pierce™ Protein A/G Magnetic Beads (Thermo Fisher Scientific). Immunoprecipitates were washed twice with high-salt buffer (50 mM Tris-HCl pH 7.4, 300 mM NaCl) followed by two additional washes with low-salt buffer (50 mM Tris-HCl pH 7.4, 150 mM NaCl). Subsequently, immunoprecipitated proteins were incubated in the presence of either RNase A (20 μg/ml, Vazyme) or RNase inhibitors (200 U/ml, Vazyme) for 5 min at 37 ℃ ^7^. The following antibodies were used for western blot analysis: anti-HNRNPA2B1 rabbit antibody, anti-HNRNPA2B1 rabbit antibody, and anti-DGCR8 rabbit antibody (all purchased from Abcam).

**m^6^A RNA immunoprecipitation assay (MeRIP)-qPCR**

The m^6^A modifications levels of individual genes were determined using the MeRIP-qPCR assay. The analysis was performed according to a previous report^8^. Briefly, total RNA was extracted from RAW264.7 cells, poly(A) RNA was first purified from 50 μg of total RNA (Dynabeads™ mRNA Purification Kit, Invitrogen), and one tenth of the RNA was saved as the input control. Pierce™ Protein A/G Magnetic Beads (Thermo Fisher Scientific, Waltham, MA, USA) were prewashed and incubated with 5 μg of anti-m^6^A antibody (Synaptic Systems, Goettingen, Germany) or rabbit IgG (Abcam). Then, the methylated mRNAs were digested in proteinase K buffer (10 mg/ml, Thermo Fisher Scientific). Further enrichment was detected by qPCR, and the corresponding m^6^A enrichment level in each sample was calculated by normalization to the input.

**RNA immunoprecipitation (RIP) assays**

RIP was conducted with the Magna RIP RNA-Binding Protein Immunoprecipitation Kit (Millipore, Billerica, MA, USA) according to the manufacturer’s instructions. Briefly, total RNA was extracted from RAW264.7 cells, and magnetic beads coated with 5 μg of specific antibodies against mouse immunoglobulin G (Millipore), METTL3 (Abcam) and/or HNRNPA2B1 (Abcam) were incubated with prepared cell lysates overnight at 4 °C. Then, the RNA protein complexes were incubated with proteinase K digestion buffer. RNA was finally extracted using phenol-chloroform RNA extraction methods. The relative expression of pri-miR-146b was determined by qPCR and normalized to the input.

***In vitro* pri-miRNA processing assays**

Pri-miRNA processing *in vitro* was performed according to a previous report^9, 10^. We used the T7-based MEGAshortscript Kit (Thermo Fisher Scientific) to induce transcription, and N6-methyl-ATP (m^6^A) (Biorbit, Cambridge, UK) was used instead of ATP in the *in vitro* transcription reaction to generate [m6A]pri-miR-146b. 293T cells were cotransfected with plasmids carrying DROSHA and DGCR8, and whole cellular lysates were harvested and then incubated with pri-miR-146b or [m6A]pri-miR-146b and pri-miR-1-1 (control) for 4 h. Total RNA puriﬁed from reaction products was analyzed by qRT-PCR. We also examined the effect of the A to T mutation at the m^6^A site of pri-miR-146b on the processing of pri-miR-146b. The primers containing the T7 promoter sequence for *in vitro* pri-miR-146b transcription are shown in Supplementary Table 1.

**Statistical analysis**

The data are presented as the mean ± SD. GraphPad Prism 8.0 software (GraphPad Software Inc., USA) was used for analysis. Statistical analysis was performed using Student’s t test, and multiple comparisons were performed through one-way ANOVA when more than two groups were compared. *p*<0.05 was considered statistically significant.

**References**

1. Ali, M.Y., Anand, S.V., Tangella, K., Ramkumar, D. & Saif, T.A. Isolation of Primary Human Colon Tumor Cells from Surgical Tissues and Culturing Them Directly on Soft Elastic Substrates for Traction Cytometry. *Journal of Visualized Experiments* (2015).

2. Peng, L. *et al.* Reprogramming macrophage orientation by microRNA 146b targeting transcription factor IRF5. *EBioMedicine* **14**, 83-96 (2016).

3. Chen, D.L. *et al.* Long non-coding RNA UICLM promotes colorectal cancer liver metastasis by acting as a ceRNA for microRNA-215 to regulate ZEB2 expression. *Theranostics* **7**, 4836-4849 (2017).

4. Kaneda, M.M. *et al.* PI3Kγ is a molecular switch that controls immune suppression. *Nature* **539**, 437-442 (2016).

5. Li, H.B. *et al.* m6A mRNA methylation controls T cell homeostasis by targeting the IL-7/STAT5/SOCS pathways. *Nature* **548**, 338-342 (2017).

6. Lan, T. *et al.* KIAA1429 contributes to liver cancer progression through N6-methyladenosine-dependent post-transcriptional modification of GATA3. *Molecular cancer* **18**, 186 (2019).

7. Alarcon, C.R. *et al.* HNRNPA2B1 Is a Mediator of m(6)A-Dependent Nuclear RNA Processing Events. *Cell* **162**, 1299-1308 (2015).

8. Li, T. *et al.* METTL3 facilitates tumor progression via an m(6)A-IGF2BP2-dependent mechanism in colorectal carcinoma. *Molecular cancer* **18**, 112 (2019).

9. Zhang, J. *et al.* Excessive miR-25-3p maturation via N(6)-methyladenosine stimulated by cigarette smoke promotes pancreatic cancer progression. *Nature communications* **10**, 1858 (2019).

10. Alarcon, C.R., Lee, H., Goodarzi, H., Halberg, N. & Tavazoie, S.F. N6-methyladenosine marks primary microRNAs for processing. *Nature* **519**, 482-485 (2015).

Supplementary Table 1. Sequences of siRNAs and primers used in this study

| Sequences of siRNAs used in this study | | |
| --- | --- | --- |
| Gene | Sense | Antisense |
| Control siRNA | UUCUCCGAACGUGGUCACGU | ACGUGACACGUUCGGAGAA |
| HNRNPA2B1 | GGCAUUGUCUAGACAAGAA | UUCUUGUCUAGACAAUGCC |
| HNRNPA2B1` | GCGGGAUCCUGCAAGCAAA | UUUGCUUGCAGGAUCCCGC |
| p110β | GGAAGCAAGUUCACAACUA | UAGUUGCGAACUUGCUUCC |
| CEBPβ1 | GCAAGAAGCCGGCCGACUA | UAGUCGGCCGGCUUCUUGC |
| CEBPβ2 | CCAAGAAGACGGUGGACAA | UUGUCCACCGUCUUCUUGG |

| Morpholino oligo sequences used in this study | |
| --- | --- |
| Negative control | CCTCTTACCTCAGTTACAATTTATA |
| METTL3 | CTCATTCCCTCCCAAGAGACCTGAA |

| Sequences of miR-146b agomirs | | |
| --- | --- | --- |
| Negative control | UUCUCCGAACGUGUCACGUTT | ACGUGACACGUUCGGAGAATT |
| miR-146b agomir | UGAGAACUGAAUUCCAUAGGCU | CCUAUGGAAUUCAGUUCUCAUU |

| Primer sequences used for real-time PCR | | |
| --- | --- | --- |
| Gene | Primer (Forward) | Primer (Reverse) |
| STAT4 | TCAGTGAGAGCCATCTTGGAGG | TGTAGTCTCGCAGGATGTCAGC |
| ICOS | GCAGCTTTCGTTGTGGTACTCC | TGTGTTGACTGCCGCCATGAAC |
| GZMK | CCATTCTCACGACTTCAGTCCG | TCACCTGGCATTTGGTCCCATC |
| IL18R | ACAACACGGACCATACGGCTGA | GTACCAGTAGAGGAAAGCAGCTG |
| CD3e | GCTCCAGGATTTCTCGGAAGTC | ATGGCTACTGCTGTCAGGTCCA |
| CXCR3 | TACGATCAGCGCCTCAATGCCA | AGCAGGAAACCAGCCACTAGCT |
| CARD11 | CACGAAGAGGATTTCACAGACGG | GCCGTGATTGACATGATGCTGC |
| METTL3 | TGATTGAGGTAAAGCGAGGTC | TCCTGACTGACCTTCTTGCTC |
| METTL14 | CTGAGAGTGCGGATAGCATTG | GAGCAGATGTATCATAGGAAGCC |
| WTAP | AGTGCCTGGAAGTTTACGCCTG | GCTTCAAGCTGTGCAATACGGC |
| FTO | TTCATGCTGGATGACCTCAATG | GCCAACTGACAGCGTTCTAAG |
| ALKBH5 | CGCGGTCATCAACGACTACC | ATGGGCTTGAACTGGAACTTG |
| HNRNPA2B1 | ACGAGAATCCCCAAAACCA | TGCAGAAGTTAGAAAGGCATTG |
| Arg1 | CTCCAAGCCAAAGTCCTTAGAG | AGGAGCTGTCATTAGGGACATC |
| YM1 | CAGGTCTGGCAATTCTTCTGAA | GTCTTGCTCATGTGTGTAAGTGA |
| IL-1β | GACGGCACACCCACCCT | AAACCGTTTTTCCATCTTCTTCTTT |
| IL12p40 | ACAGCACCAGCTTCTTCATCAG | TCTTCAAAGGCTTCATCTGCAA |
| IL-6 | GTAGCTATGGTACTCCAGAAGAC | ACGATGATGCACTTGCAGAA |
| INOS | CCAGCACTTCACCCATCAGTT | AAGGCGCAGTTTATGTTGTCTGT |
| TNFα | GCCACCACGCTCTTCTGTCT | GGTCTGGGCCATAGAACTGATG |
| IL-10 | GCTCTTGCACTACCAAAGCC | CTGCTGATCCTCATGCCAGT |
| CCL2 | CCACAACCACCTCAAGCACT | TAAGGCATCACAGTCCGAGTC |
| CEBPβ | GGAGACGCAGCACAAGGT | AGCTGCTTGAACAAGTTCCG |
| p110β | AGAAGCTGGCTTGGACCTG | CAGAGCGATCTCCTGTTGCT |
| GAPDH | TCCCACTCTTCCACCTTCGATGC | GGGTCTGGGATGGAAATTGTGAGG |

| Sequences of primers for used *in vitro* transcription | | |
| --- | --- | --- |
| Gene | Primer (Forward) | Primer (Reverse) |
| Pri-miR-146b | TAATACGACTCACTATAGGGGGTAAG  AACAGCCCACAGAAG | ATGCATCTAGATATCGGATCCCCA  GAAGAAACCCCATTTCAAAA |
| Pri-miR  -1-1 | TAATACGACTCACTATAGGGGGTTTC  TTAGAGTCTGGGATG | GTGGCAGAACAATGCCAACAGG  GACAGGGACACAGCCGGTAC |


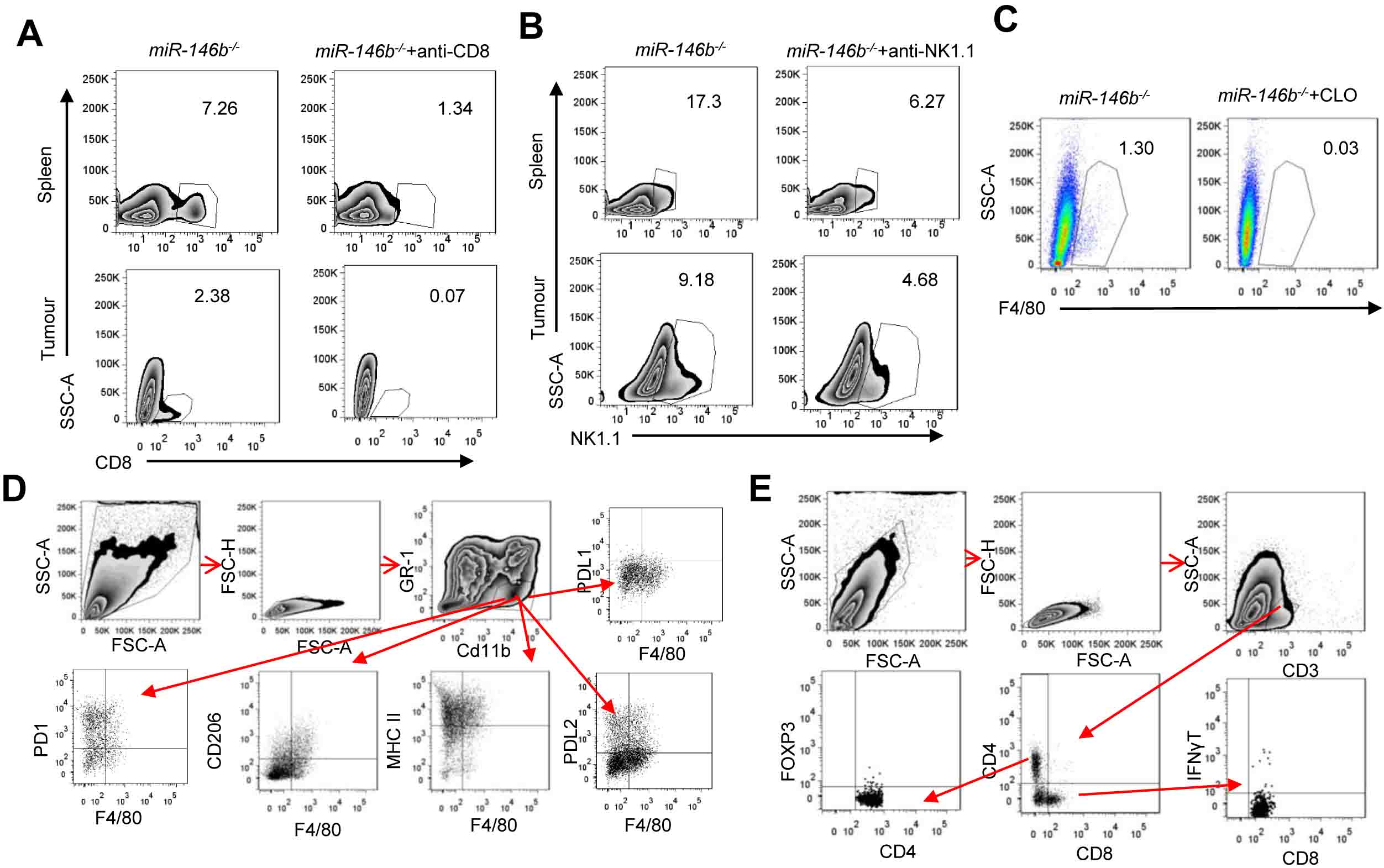


**Supplementary Figure 1.** Validation of CD8-, NK- or macrophage depletion *in vivo*. WT and *miR-146b^-/-^* mice were injected subcutaneously with MC38 cells and treated with clodronate and a CD8- or NK-depleting antibody. **(A-C)** Flow cytometry analysis of the frequencies of CD8^+^ cells, NK cells and macrophages in tumors. **(D)** Gating strategy for flow cytometric analysis of immune cell populations in tumor tissue. Doublets were removed, and TAMs were assessed as CD11b^+^Gr1^-^F4/80^+^CD206^+^ TAMs and M1 macrophages (CD11b^+^Gr1^-^F4/80^+^MHC II^+^). **(E)** Doublets were removed. Then, CD4^+^ T cells were assessed as CD3^+^CD4^+^ cells, and CD8^+^ T cells were assessed as CD3^+^CD8^+^ cells. Treg cells were assessed as CD3^+^CD4^+^FOXP3^+^ cells, and CTLs were assessed as CD3^+^CD8^+^IFNγ^+^ cells.


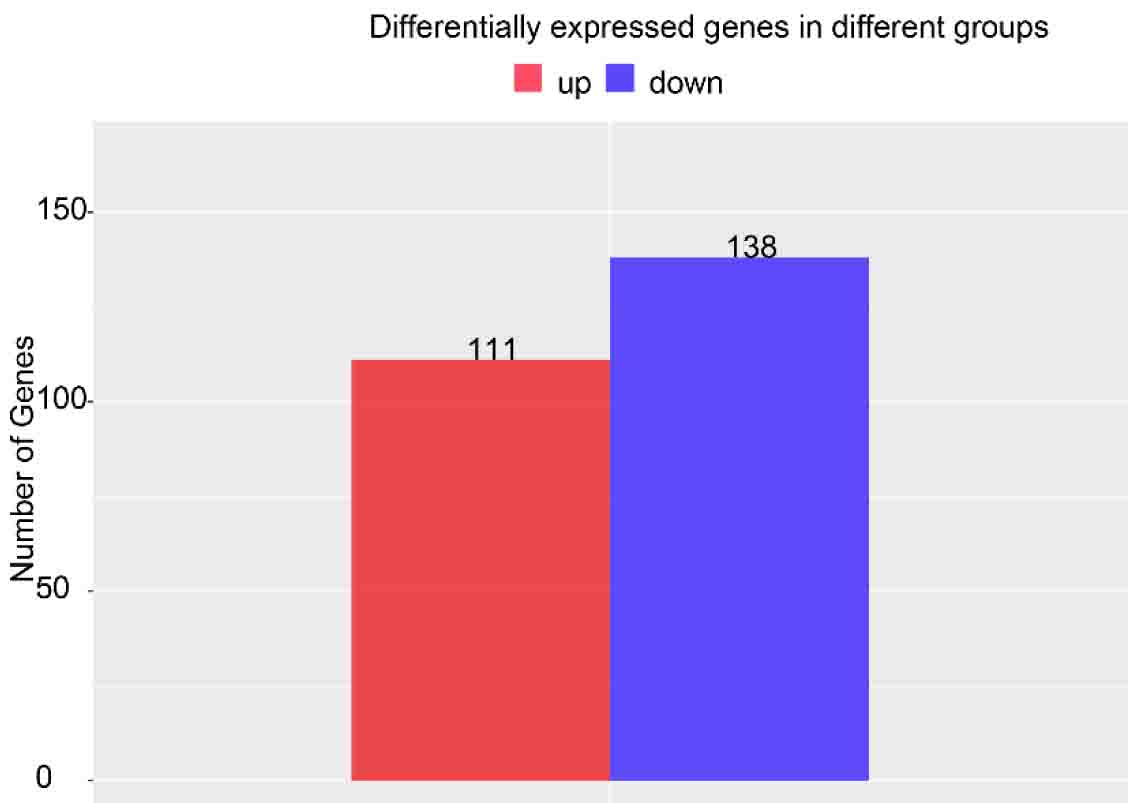


**Supplementary Figure 2.** WT and *miR-146b^-/-^* mice were implanted with MC38 cells for 3 weeks. The differences in gene expression in *miR-146b^-/-^* mice compared with WT mice were identified. A total of 111 downregulated and 138 upregulated genes were differentially expressed in tumor tissue in *miR-146b^-/-^* mice.


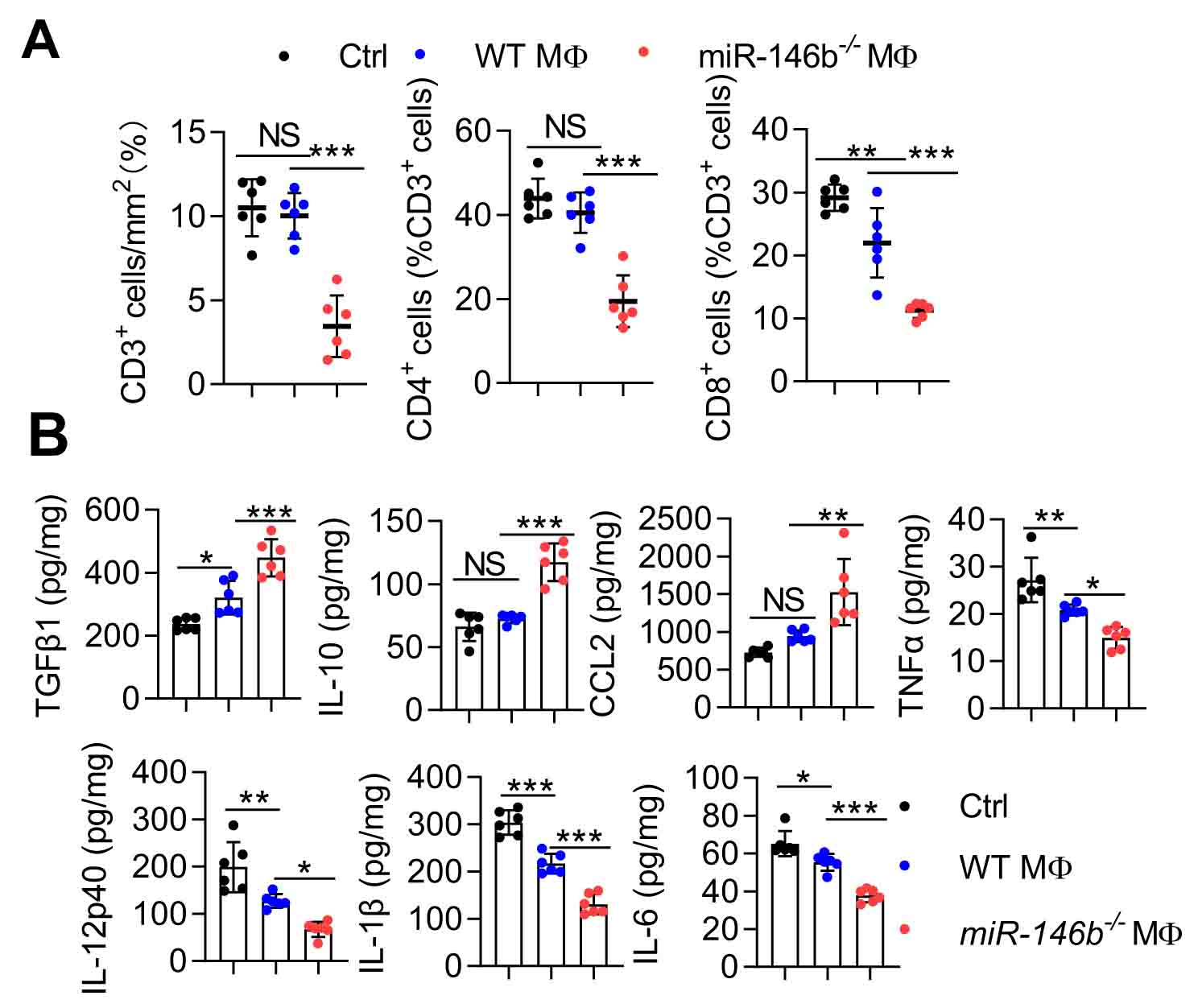


**Supplementary Figure 3.** miR-146b^-/-^ macrophages promote immune suppression. **(A)** Flow cytometric analysis of T-cell populations in tumors from mice implanted with *in vitro* cultured M2 macrophages. **(B)** TGFβ1, IL-10, MCP-1, TNFα, IL-6, IL-1β and IL-12p40 protein expression in tumors from mice implanted with *in vitro* cultured M2 macrophages was determined by ELISA. The data represent the mean ± SD. **p*<0.05; ***p*<0.01; ****p*<0.001; NS, not signiﬁcant.


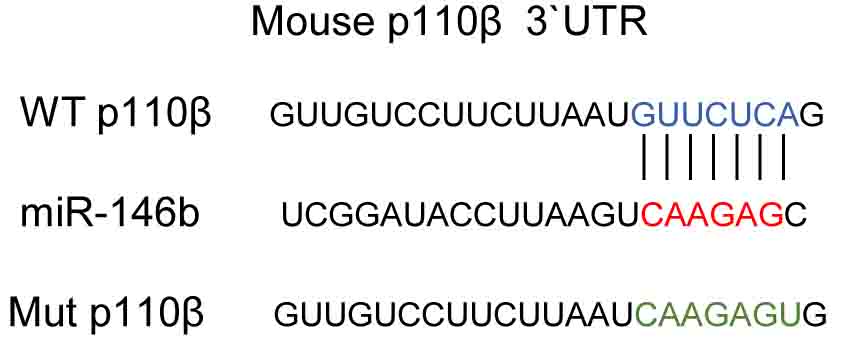


**Supplementary Figure 4.** Schematic representation of wild-type (WT) and mutant (Mut) p110β 3′UTR luciferase reporter constructs (upper).


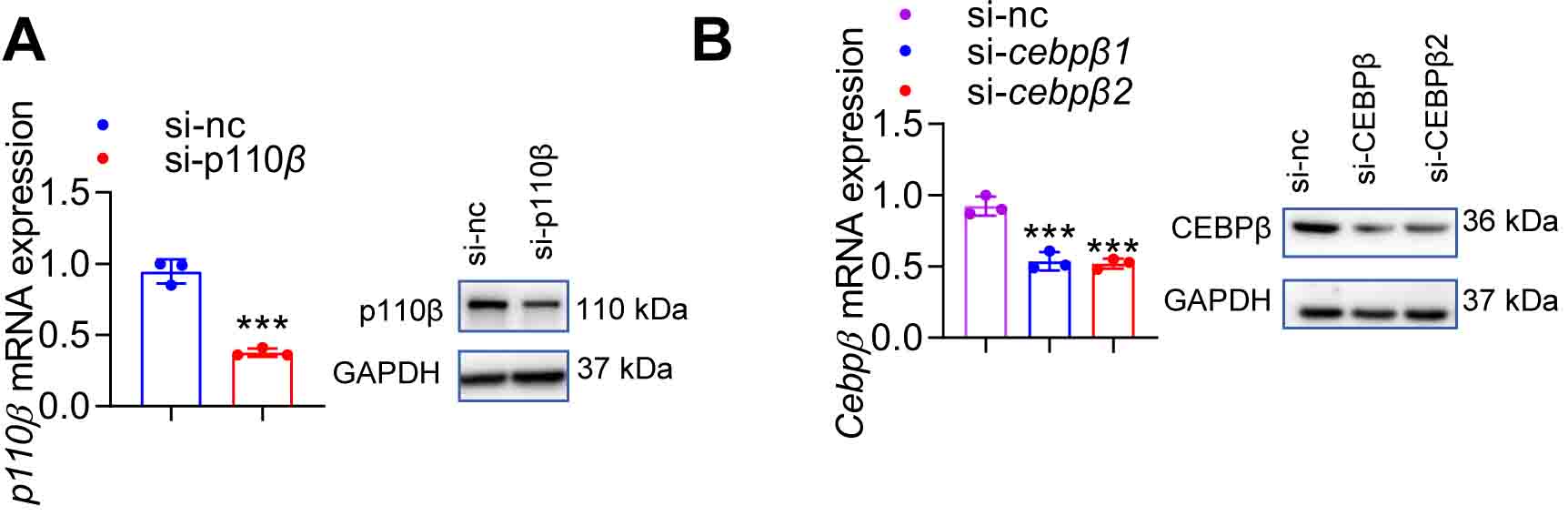


**Supplementary Figure 5.** **(A)** *miR-146b^-/-^* BMDMs were transfected with si-*cebpβ* for 12 h. The mRNA and protein levels of CEBPβ were determined by qPCR and western blotting respectively. **(B)** *miR-146b^-/-^* cells were transfected with si-*p110β*. The mRNA and protein levels of p110β were determined by qPCR and western blotting respectively. The data represent the mean ± SD. ****p*<0.001.


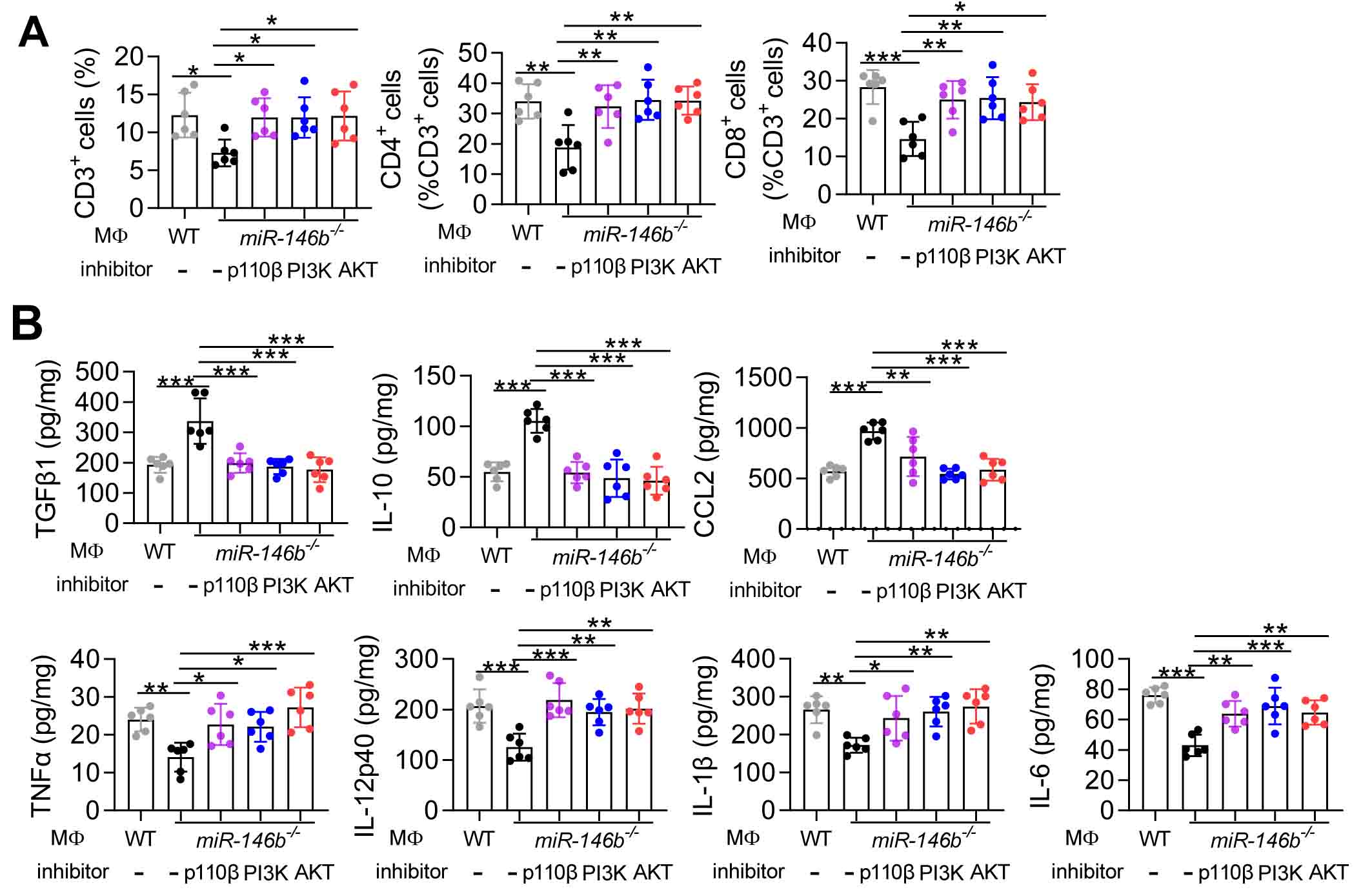


**Supplementary Figure 6.** MC38 cells were mixed with WT M2 macrophages and *miR-146b^-/-^* M2 macrophages pretreated with the p110β inhibitor (TGX221), the AKT inhibitor (GSK2141795) or the PI3K inhibitor (LY294002). **(A)** Flow cytometric analysis of T-cell populations in tumors (n=6). **(B)** TGFβ1, IL-10, MCP-1, TNFα, IL-6, IL-1β and IL-12p40 protein expression in tumors was determined by ELISA (n=6). The data represent the mean ± SD. **p*<0.05; ***p*<0.01; ****p*<0.001; NS, not signiﬁcant.


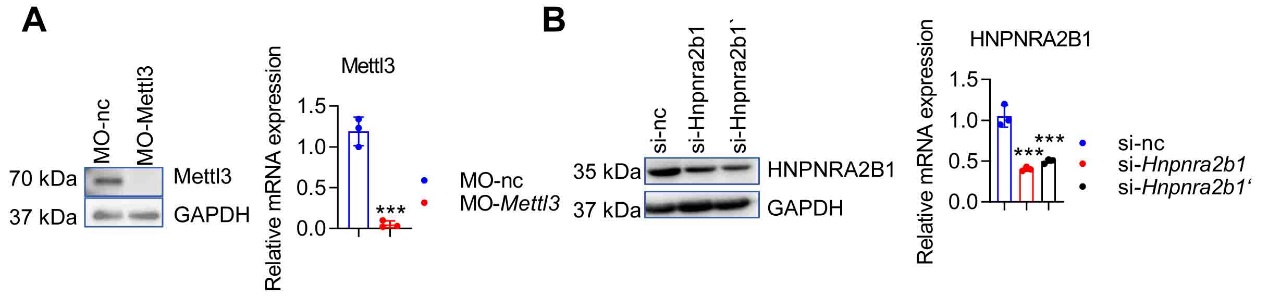


**Supplementary Figure 7. (A)** WT BMDMs were transfected with MO-*Mettl3*. Mettl3 mRNA and protein levels were determined by qPCR and western blotting respectively. **(B)** WT BMDMs were transfected with si-*Hnrnpa2b1*. The mRNA and protein levels of HNRNPA2B1 were determined by qPCR and western blotting respectively. The data represent the mean ± SD. ****p*<0.001.


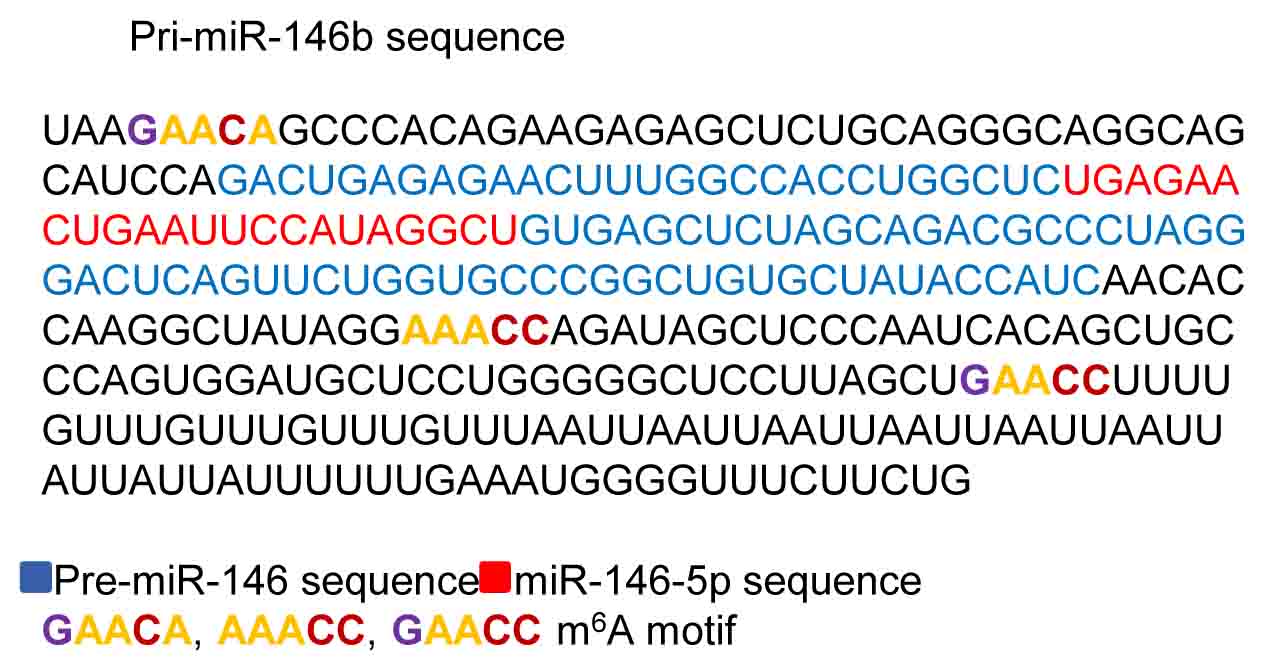


**Supplementary Figure 8.** The sequences of pre-miR-146b and miR-146b-5p are highlighted in different colors, and the m^6^A motif is located at the putative splicing site.


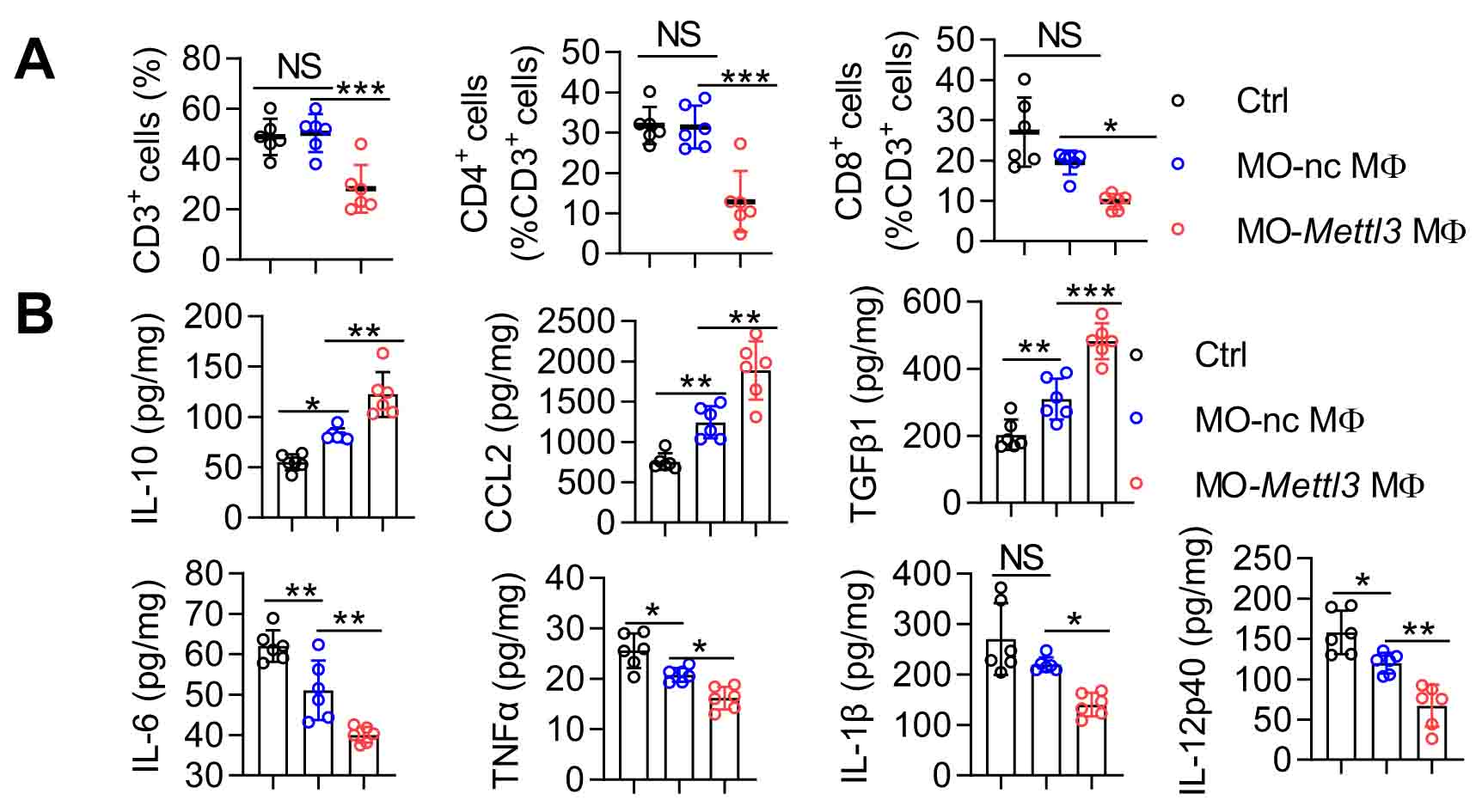


**Supplementary Figure 9.** BMDMs from WT mice were transfected with MO followed by treatment with IL-4 plus IL-10. WT mice were implanted with MC38 cells mixed with *in vitro* cultured M2 macrophages (n=6). **(A)** FACS analysis of T cells in tumors. **(B)** Cytokine protein expression in tumors was analyzed by ELISA. The data represent the mean ± SD. **p*<0.05; ***p*<0.01; ****p*<0.001; NS, not signiﬁcant.
